# SpyB, a Small Heme-Binding Protein, Affects the Composition of the Cell Wall in *Streptococcus pyogenes*

**DOI:** 10.3389/fcimb.2016.00126

**Published:** 2016-10-13

**Authors:** Rebecca J. Edgar, Jing Chen, Sashi Kant, Elena Rechkina, Jeffrey S. Rush, Lennart S. Forsberg, Bernhard Jaehrig, Parastoo Azadi, Veronika Tchesnokova, Evgeni V. Sokurenko, Haining Zhu, Konstantin V. Korotkov, Vijay Pancholi, Natalia Korotkova

**Affiliations:** ^1^Department of Molecular and Cellular Biochemistry, University of KentuckyLexington, KY, USA; ^2^Department of Pathology, Ohio State UniversityColumbus, OH, USA; ^3^Department of Microbiology, University of WashingtonSeattle, WA, USA; ^4^Complex Carbohydrate Research Center, University of GeorgiaAthens, GA, USA

**Keywords:** SpyA, SpyB, heme, ADP-ribosyltransferase, Group A carbohydrate, *Streptococcus pyogenes*, cell wall

## Abstract

*Streptococcus pyogenes* (Group A *Streptococcus* or GAS) is a hemolytic human pathogen associated with a wide variety of infections ranging from minor skin and throat infections to life-threatening invasive diseases. The cell wall of GAS consists of peptidoglycan sacculus decorated with a carbohydrate comprising a polyrhamnose backbone with immunodominant N-acetylglucosamine side-chains. All GAS genomes contain the *spyBA* operon, which encodes a 35-amino-acid membrane protein SpyB, and a membrane-bound C3-like ADP-ribosyltransferase SpyA. In this study, we addressed the function of SpyB in GAS. Phenotypic analysis of a *spyB* deletion mutant revealed increased bacterial aggregation, and reduced sensitivity to β-lactams of the cephalosporin class and peptidoglycan hydrolase PlyC. Glycosyl composition analysis of cell wall isolated from the *spyB* mutant suggested an altered carbohydrate structure compared with the wild-type strain. Furthermore, we found that SpyB associates with heme and protoporphyrin IX. Heme binding induces SpyB dimerization, which involves disulfide bond formation between the subunits. Thus, our data suggest the possibility that SpyB activity is regulated by heme.

## Introduction

Group A *Streptococcus* (GAS; *Streptococcus pyogenes*) is an important human pathogen that causes a variety of diseases including minor skin and throat infections, such as impetigo and pharyngitis, and life-threatening invasive infections, such as streptococcal toxic shock syndrome and necrotizing fasciitis. Worldwide GAS infections account for more than 600 million cases of pharyngitis and more than 650,000 cases of invasive disease, with >500,000 deaths annually (Carapetis et al., 2005). Although the pathogen is susceptible to antibiotic therapy, severe invasive GAS infections pose formidable challenges to public health.

GAS possesses a wide array of virulence factors that contribute to its ability to cause infections, ranging from secreted toxins, hemolysins, and adhesins, to mechanisms of sequestering essential nutrients, as well as sensing mechanisms for avoiding or subverting host defenses. All GAS genomes possess an operon consisting of two genes, *spyA* and *spyB* (Korotkova et al., 2012). SpyA is an ADP-ribosyltransferase that catalyzes the covalent transfer of an ADP-ribose moiety of NAD to target proteins. Deletion of *spyA* in the M1T1 clone of GAS resulted in increased bacterial uptake by macrophages (Lin et al., 2015). Moreover, the SpyA-deficient mutant demonstrated impaired bacterial clearance in the mouse model of GAS infection (Lin et al., 2015). These observations suggest a potential role for SpyA in GAS invasive infections. Based on SpyA homology to C3-like ADP-ribosyltransferase toxins, the protein has been considered as a toxin targeting host processes (Coye and Collins, 2004; Hoff et al., 2011; Icenogle et al., 2012; Lin et al., 2015). However, we have shown that SpyA is an extracellular membrane-associated protein that is not released into the extracellular space (Korotkova et al., 2012). This observation suggests that SpyA is involved in the modification of GAS extracellular proteins. Immediately upstream from *spyA*, a small gene *spyB* was identified that encodes a 35 amino acid-peptide (Korotkova et al., 2012). The SpyB N-terminus is predicted to fold into an amphipathic α-helix, a structural motif that targets a protein to negatively charged phospholipid membranes. Moreover we identified SpyB as a potential ADP-ribosylated substrate of SpyA (Korotkova et al., 2012).

This study was initiated to understand the function of SpyB in GAS. Since *spyB* and *spyA* are located in the same operon and are likely to be functionally connected, understanding the role of SpyB in GAS biology will pay dividends for the characterization of SpyA. Here we report that SpyB is a heme binding protein. Phenotypic characterization and cell wall structure analysis of a *spyB* deletion mutant suggests that SpyB functions to modulate cell wall composition in GAS.

## Materials and methods

### Bacterial strains and growth conditions

All plasmids, strains and primers used in this study are listed in Tables S1–S3 in the Supplemental Material. The GAS strains used in this study were MGAS5005, an M1-serotype strain isolated from the cerebral fluid of a patient with bacterial meningitis (Sumby et al., 2005) and the *spyA* isogenic mutant 5005Δ*spyA* (Hoff et al., 2011). Whole-genome sequencing was performed for 5005Δ*spyA* to confirm the deletion in *spyA* and the absence of additional mutations. The genome sequencing was conducted essentially as described in Price et al. (2013) using Illumina Genome Analyzer IIx. Illumina whole-genome sequence data sets were aligned against the chromosome of a published MGAS5005 reference genome (GenBank accession no. CP000017) (Sumby et al., 2005) using the short-read alignment component of the Burrows-Wheeler Aligner. Mutations were identified as described in Price et al. (2013).

GAS cultures were grown in Todd-Hewitt broth supplemented with 0.2% yeast extract (THY), or on THY agar plates. When indicated, strains were cultured in a chemically defined medium [CDM (Chang et al., 2011)] supplied with 1% glucose. GAS strains were incubated without aeration at 37°C. *Escherichia coli* strains were grown in Luria-Bertani (LB) medium or on LB agar plates at 37°C. When required, antibiotics were included at the following concentrations: ampicillin at 100 μg ml^−1^ for *E. coli*; chloramphenicol at 10 μg ml^−1^ for *E. coli* and 5 μg ml^−1^ for GAS.

To analyze the effect of hemin on growth of GAS strains, frozen aliquots of GAS strains were prepared for experiments. To make frozen aliquots GAS strains cultured overnight in THY medium were diluted 1:100 into fresh THY medium and allowed to grow to mid-logarithmic phase (OD_600_ = 0.7). Bacteria were collected by centrifugation, washed twice with PBS and re-suspended in PBS with 15% glycerol. The volume of PBS/glycerol was equal to a half volume of THY medium used for culturing. The aliquots were frozen and stored at −80°C. On experiment days, the aliquots were thawed, diluted 1:20 into THY medium. Bacteria were grown in 12-ml THY medium tightly sealed in 15-ml conical tubes without aeration at 37°C. Hemin chloride (Sigma) from stock solution prepared in DMSO was added to the growth media at a concentration of 2 μM.

### DNA techniques

Plasmid DNA was isolated from *E. coli* by commercial kits (Qiagen) according to the manufacturer's instructions and used to transform *E. coli* and GAS strains. Plasmids were transformed into GAS by electroporation as described previously (Hoff et al., 2011). Chromosomal DNA was purified from GAS as described in Caparon and Scott (1991). Constructs containing mutations were identified by sequence analysis. Primers for site-directed mutagenesis are listed in Table S3. All constructs were confirmed by sequencing analysis (Eurofins MWG Operon).

### Plasmid and strain construction

#### Construction of the *spyB* deletion mutant

For construction of the 5005Δ*spyB* mutant, MGAS5005 chromosomal DNA was used as a template for amplification of a 1.4 kbp PCR product. The primer pair spyB-BamHI-f and spyB-XhoI-r (Table S2) was used to amplify a DNA region flanking either side of the *spyB* upstream region. The PCR product was digested with *Bam*HI and *Xho*I and ligated into *Bgl*II/*Sal*I-digested plasmid pBBL740 (Table S1). The integrational plasmid pBBL740 does not have a replication origin that is functional in GAS, so the plasmid can be maintained only by integrating into the GAS chromosome through homologous recombination. To create an in frame deletion in *spyB*, the resultant plasmid pBBL740*spyB* was employed for site-directed mutagenesis using the primer pair listed in Table S3. The resulting plasmid, pBBL740Δ*spyB*, was transformed into MGAS5005, and chloramphenicol resistant colonies were selected on THY agar plates. Five randomly selected colonies, that had the first crossover, were grown in liquid THY without chloramphenicol for ≥5 serial passages. Several potential double crossover mutants were selected as previously described (Treviño et al., 2013). The deletion in each mutant was confirmed by sequencing a PCR fragment. Whole-genome sequencing was performed for strains to confirm the deletion and the absence of additional mutations. A schematic representation of the construction of the 5005Δ*spyB* mutant is shown in Figure S1.

#### Complementation of the 5005Δ*spyB* mutant in *cis* with *spyB* (5005Δ*spyB spyB*^+^)

To construct the plasmid for *in cis* complementation of the 5005Δ*spyB* mutant, the pBBL740*spyB* plasmid was employed. A synonymous mutation was introduced in Arg 9 of SpyB by site-directed mutagenesis using the primer pair listed in Table S3. This mutation was necessary to distinguish the complemented strain from the wild-type (WT) strain. The plasmid was designated p*spyBs*. The plasmid was transformed into the 5005Δ*spyB* strain, and chloramphenicol resistant colonies were selected on THY agar plates. Double crossover mutants were selected as described above. The replacement of truncated *spyB* with the *spyB* containing Arg 9 synonymous mutation was confirmed by sequencing a PCR fragment. The complementation did not show a polar effect on *spyA* transcription (Figure S2A). A schematic representation of the construction of the 5005Δ*spyB spyB*^+^ strain is shown in Figure S1.

#### Construction of the plasmid for *E. coli* expression of MBP fusion with *spyB*

To create a vector for expression of MBP fusions, *malE* was amplified from *E.coli* chromosomal DNA using the primer pair malE-NdeI-f and malE-NcoI-r (Table S2). The PCR product was digested with *Nde*I and *Nco*I, and ligated into *Nde*I/*Nco*I-digested pET-22b(+) vector. The resultant plasmid, pmalE, contains *malE* followed by a multiple cloning site and the sequence encoding a His-tag. A DNA fragment containing *spyB* was amplified from MGAS5005 chromosomal DNA. A TEV protease recognition site at the C-terminus of *spyB* was introduced with the reverse primer, using PCR amplification with the primer pair spyB-NcoI-f and spyB-TEV-XhoI-r (Table S2). The PCR product was digested with *Nco*I and *Xho*I, and was ligated into pmalE digested with the same enzymes. The resultant plasmid, pmalEspyB, contained *spyB* fused at the N-terminus with *malE* and at the C-terminus with a TEV protease recognition site followed by a His-tag sequence. Mutations in *spyB* were introduced in the pmalEspyB plasmid by site directed mutagenesis using primers listed in Table S3. The resultant plasmids were transferred into competent *E. coli* Rosetta (DE3) (Novagen) using the manufacturer's protocol.

### Quantitative RT-PCR (qRT-PCR)

Total RNA was isolated from GAS grown to mid-exponential phase (OD_600_ = 0.6) using the miRNeasy kit (Qiagen) according to manufacturer's instructions. Contaminating genomic DNA was removed from RNA samples with Turbo DNase (Applied Biosystems). cDNA for qRT-PCR was obtained as described in Falaleeva et al. (2014). The efficacy of the DNase step was controlled for by including a reverse transcriptase negative control. qRT-PCR was employed to examine relative mRNA abundance of *spyA* with the house keeping gene *plr* used as an internal standard for normalization. qRT-PCR reactions were performed using a Mx3005P qPCR system (Agilent Technologies) under standard reaction conditions. The reaction mixture contained Power SYBR green PCR Master Mix (Applied Biosystems), cDNA and primers spyArt-f and spyArt-r (Table S2). *plr* was analyzed in each cDNA reaction using the pair of primers plr-f and plr-r (Table S2). The expression levels of *spyA* in each condition tested were normalized to the levels of *plr* transcript. No-template and no RT controls were included for each primer set and template. Data is reported as the mean values of fold change in mRNA levels in the mutants relative to those of MGAS5005 WT strain. Each experiment was performed in triplicate, and mean values ± standard deviations (*SD*s) are shown.

### Purification of MBP-SpyB fusion

The MBP-SpyB protein was expressed in *E. coli* Rosetta (DE3) harboring pmalEspyB plasmid. The frozen cells were resuspended in 20 mM Tris pH 7.5, 300 mM NaCl at 4°C. The suspension was passed twice through a microfluidizer cell disrupter and the lysate centrifuged for 45 min at 17,000 × g. The protein was purified by Ni-NTA affinity chromatography and dialyzed overnight at 4°C in 20 mM Tris pH 7.5, 300 mM NaCl in the presence of TEV protease. Cleavage with TEV protease leaves the first six residues of the TEV site (ENLYFQ) at the C-terminus of MBP-SpyB.

The cleaved protein solution was purified by Ni-NTA affinity chromatography. The removed His-tag binds to the resin while MBP-SpyB, without His-tag, immediately elutes from the column. The protein was concentrated to around 10 mg mL^−1^, treated with 1 mM DTT and purified on the size-exclusion chromatography column Superose 6 10/300, pre-equilibrated with 20 mM HEPES pH 7.5, 100 mM NaCl, 1 mM DTT, and 2 mM maltose. The protein was present as a monomer and heme-bound dimer. The protein fractions corresponding to the monomeric form were pooled, concentrated and used for reconstitution with hemin.

### Purification of MBP

MBP protein was expressed in *E. coli* Rosetta (DE3) harboring pKV1111 plasmid (Dunstan et al., 2013). The protein was purified by Ni-NTA affinity chromatography, followed by size-exclusion chromatography on a Superose 6 10/300 column, as for MBP-SpyB.

### Heme reconstitution

Hemin chloride was dissolved in 0.1 M NaOH at 10 mM concentration and diluted in protein buffer as desired. MBP-SpyB monomer was reconstituted with hemin in the presence of 1 mM DTT at room temperature at a protein:hemin ratio of 1:4 for 1 h. The sample was then loaded onto a Sephadex G-25 column and the holoprotein was eluted with 20 mM Tris-HCl and 100 mM NaCl, pH 7.5. The holoprotein was further purified on the Superose 6 size-exclusion chromatography column, as described above. The protein fractions corresponding to the holoprotein dimer form were pooled, concentrated and used for experiments.

### Tetramethylbenzidine (TMBZ) heme staining

TMBZ staining of native-PAGE to detect for the presence of bound hemin was carried out according to the protocol described in Thomas et al. (1976). Briefly, 6.3 mM TMBZ in methanol was freshly made and mixed with 0.25 mM sodium acetate pH 5.0 in a ratio of three parts to seven. The gel was incubated with the TMBZ solution in the dark for 1–2 h. The bands were developed with the addition of 30 mM H_2_O_2_ for 30 min. The gel was fixed with three washes of isopropanol and 0.25 mM sodium acetate pH 5.0 in a ratio of three parts to seven.

### Tryptophan fluorescence quenching assay

The SpyB peptide was chemically synthesized (GenScript, NJ). The MBP-SpyB, MBP-SpyB C7A/C13A, MBP-SpyB C7A/C13A/C30A/C35A monomers and MBP were purified as described above. Hemin binding was monitored by fluorescence quenching of the tryptophan residues of SpyB at 20°C using a PerkinElmer LS 55 Fluorescence spectrometer as described in Shepherd et al. (2007). Hemin (prepared as described above, in various dilutions) or protoporphyrin IX (prepared as a 10 mM stock in ddH_2_O and diluted in protein buffer) were added to 50 μl of protein solution (5 μM, in 20 mM HEPES, 100 mM NaCl, and 1 mM DTT, pH 7.5) in 50 μl aliquots. The excitation wavelength was 280 nm and the emission wavelength was from 300 to 500 nm. The dissociation constant (K_D_) was calculated using the change in fluorescence (ΔF) and concentration of porphyrin (hemin or protoporphyrin IX; [porphyrin]) using SigmaPlot v12.3 and the following equation:

$$\begin{array}{l} \Delta \text{F}=\frac{\Delta {\text{F}}_{\text{max}}\times \left[\text{porphyrin}\right]}{{\text{K}}_{\text{D}}+\left[\text{porphyrin}\right]}. \end{array}$$

The Stern-Volmer constant (K_sv_) was calculated to determine whether porphyrin binding was dynamic or static. K_sv_ was calculated using [porphyrin] and fluorescence intensities (F) according to the following equation:

$$\begin{array}{l} {\text{F}}_{0}/\text{F}=1+{\text{K}}_{\text{sv}}\left[por\text{porphyrin}\right]. \end{array}$$

### UV-visible absorption spectroscopy

Absorbance spectrums were recorded between 300 and 700 nm for 50 μM hemin, and 50 μM hemin mixed with 2.5 μM MBP-SpyB or MBP, purified as described above. After mixing in the presence of 1 mM DTT, the spectrums were immediately recorded on a SpectraMax M5 (Molecular Devices).

### Pyridine hemochrome assay

Protein concentrations were determined using a modified BCA protein assay kit (Pierce, Rockford, IL) with bovine serum albumin (BSA) as a standard. The heme to protein ratio was measured by pyridine hemochrome assay according to the method (Berry and Trumpower, 1987). Briefly, 100 μl of protein sample in Tris-HCl buffer was mixed with 10 μl 1 N NaOH, 23 μl pyridine, and ~2 mg sodium dithionite. The optical absorption spectrum was immediately recorded between 280 and 700 nm using a SpectraMax M5 (Molecular Devices) spectrophotometer. The heme content was calculated using the extinction coefficient ε_418_ = 191.5 mM^−1^ cm^−1^.

### Dissociation of hemin from Holo-MBP-SpyB

The rate of hemin dissociation from SpyB was determined using the procedure described in Ortiz de Orué Lucana et al. (2014). Briefly, 5 μM MBP-SpyB was incubated with 20 μM hemin in 20 mM Tris-HCl, pH 7.5, 1 mM DTT at 4°C for 1 h. Free hemin was removed and MBP-SpyB:hemin holoprotein was collected by size-exclusion chromatography under the same conditions described above. The holoprotein was diluted to 4 μM and incubated with 4 μM apo-myoglobin for 20 min at 25°C with absorbance readings at 408 nm every 5 s. GraphPad Prism v 4.0 software was used to calculate the dissociation rate constant (*k*_*off*_) from the change in absorbance at 480 nm. Apo-myoglobin was generated from hemoglobin by methyl ethyl ketone extraction as described in Ascoli et al. (1980).

### Dynamic light scattering

All samples were filtered through a 0.22-μm filter before analysis. Dynamic light scattering was conducted on a DynaPro Nanostar (Wyatt Technologies). MBP-SpyB was analyzed at a final concentration of 40 μM. Measurements were taken over a 54 s time period with 10 replicates at room temperature. The samples were illuminated with a HeNe laser (633 nm) and experiments were conducted at a scattering angle of 90°. The associated DYNAMICS analysis software was used to determine average radii.

### Analysis of SpyB solubility

Synthetic SpyB (30 μM) was mixed with hemin in different molar ratios. The samples were centrifuged at maximum speed for 5 min to collect the supernatant and pellet. Samples were dissolved in SDS-PAGE loading buffer and resolved on 16% Tris-Tricine gel.

### Identification of SpyB disulfide bonds by mass-spectrometry (MS)

MBP-SpyB holoprotein dimer was purified as described above and split into three. One sample was first reduced (10 mM DTT, 30 min, 56°C), then alkylated with 50 mM iodoacetamide (IAA) for 30 min in the dark at room temperature. The sample was digested overnight with trypsin at a 1:100 (w/w) enzyme to substrate ratio for 12 h at 37°C. After these treatments all free cysteine residues and residues that participate in disulfide bond formation should be found alkylated with IAA.

The second sample was alkylated only (50 mM IAA, for 30 min in the dark at room temperature), and then processed as described above. After these treatments, all free cysteine residues should be found alkylated with IAA.

For the assignment of disulfide bonds, the final holoprotein dimer sample was neither reduced nor alkylated. The sample was dialyzed and digested as described above. Tryptic fragments with disulfide bonds that disappeared after the reduction/alkylation treatment were identified by comparison of the two LC-MS/MS runs (with and without reduction/alkylation). LC-MS/MS analysis was performed using an LTQ-Orbitrap mass spectrometer (Thermo Fisher Scientific) coupled with an Eksigent Nanoflex cHiPLC system (Eksigent) through a nanoelectrospray ionization source. The LC-MS/MS data were subjected to database searches for protein identification using Proteome Discoverer software V. 1.3 (Thermo Fisher Scientific) with a local MASCOT search engine. A custom database containing the protein of interest was used.

### Phenotype microarrays

Growth phenotypes of MGAS5005 and the 5005Δ*spyA* and 5005Δ*spyB* mutants were assessed using the Biolog plates at Biolog's PM Services facility. A total of 20 96-well PM plates constituting eight metabolic panels (PM1–PM8); and 12 sensitivity panels (PM9–PM20) were used in this study. Two replicates were conducted for each strain. Standard PM testing protocols are described in http://www.biolog.com.

### Microscopy

GAS strains were harvested from the exponential growth phase (OD_600_ = 0.5) by centrifugation. Bacteria were washed with phosphate-buffered saline (PBS) and subjected to light (Gram stain) and fluorescent microscopy. For fluorescent microscopy, GAS strains were incubated with vancomycin-BODIPY for 30 min, washed and spread on glass slides, and imaged using a Nikon Eclipse E600 as described in Kant et al. (2015). For transmission and scanning electron microscopy the bacterial pellets were washed once with PBS and then fixed with freshly made 2.5% glutaraldehyde and 4% paraformaldehyde, in 0.1 M cacodylate buffer, pH 7.4. The fixed samples were processed at the Ohio State University Microscopy and Imaging Facility and imaged by field-emission gun-equipped scanning (Nova NanoSEM 400, FEI) or cryo-capable transmission electron microscopy (Tecnai G2 Spirit, FEI) as described in Agarwal et al. (2011) and Kant et al. (2015).

### Analysis of GAS resistance to β-lactam antibiotics

Aliquots of frozen bacteria were used to analyze GAS resistance to β-lactam antibiotics, and were prepared as described above. On experiment days, the aliquots were thawed, diluted 1:20 into CDM and grown to OD_600_ = 0.5. Bacteria were collected by centrifugation and re-suspended in PBS (10^−2^ dilution). For the antibiotics challenge, 10 μl of bacterial solution was spotted on THY plates unmodified or supplemented with 0.05–0.25 μM cefoperazone, 130–920 μM methicillin or 30–140 μM ampicillin. The experiment has been conducted at least three times. The minimum inhibitory concentration (MIC) of an antibiotic is defined as the lowest concentration of the compound that completely inhibits bacterial growth on plates.

### Detection of PBPs

Bacteria from 10 ml of exponential culture at an OD_600_ = 0.5 were harvested by centrifugation. The pellet was washed in PBS and re-suspended in PBS with 0.3, 0.45, or 0.6 μM cefoperazone. A reference sample was re-suspended in PBS without the antibiotic. After 30 min of incubation at room temperature, the bacteria were pelleted, washed in PBS, and re-suspended in PBS with 15 μM Bocillin FL (Boc-FL). After 30 min incubation at room temperature, the bacteria were pelleted and washed in PBS. To digest the GAS cell wall, the pellet was re-suspended in PBS supplemented with PlyC lysin as described in Raz and Fischetti (2008) and incubated for 45 min at 37°C. The protoplasts were collected by centrifugation at 3,000 × g for 10 min. The cells were lysed by treatment with BugBuster Protein Extraction Reagent (Novagen) according to the manufacturer's protocol. The protein concentration was measured with a NanoDrop 1000 spectrophotometer. The protein concentration was adjusted to 1.5 mg ml^−1^ and mixed with 6 × SDS-PAGE loading buffer. The samples were analyzed on 4–12% SDS-PAGE gel as described in Kocaoglu et al. (2015). Densitometry analysis was performed using ImageJ software.

### Analysis of PlyC sensitivity

Strains cultured overnight in THY medium were diluted 1:10 into fresh THY medium in a 96 well plate and allowed to grow for 2 h. Fresh plates containing 100 μL of THY and serial dilutions of PlyC (0–125 ng mL^−1^) were inoculated with 100 μL of culture (giving a final maximum concentration of 62.5 ng mL^−1^ PlyC). The cultures were incubated for 1 h at 37°C, with OD_600_ measurements obtained at 0 and 1 h. The percentage change in OD_600_ was calculated from three biological replicates.

### Binding of wheat germ agglutinin (WGA) to bacteria

Cells were collected from exponential growing cultures (OD_600_ = 0.6), washed three times with BSA-saline solution (0.5% BSA, 0.15 M NaCl) and resuspended in BSA-saline solution prior to a 30 min blocking step at 37°C with mixing. Alexa Fluor 555-WGA was subsequently added to a final concentrations of 0, 12.5, 25, 50, 100 μg ml^−1^. After 60 min of incubation at 37°C with mixing, the cells were centrifuged, washed twice, and resuspended in BSA-saline. Bound Alexa Fluor 555-WGA was quantified in a fluorimeter SpectraMax M5 (Molecular Devices) using an excitation of 544 nm and emission of 590 nm.

### Analysis of total carbohydrates

Bacteria from 15 ml of exponential culture at an OD_600_ = 0.5 were harvested by centrifugation. The pellet was washed five times in water and subjected to analysis of total carbohydrate content by the phenol-sulfuric acid method (DuBois et al., 1956). The BCA protein assay was used to quantify total protein and normalize samples.

### Isolation of cell wall

GAS cell wall was prepared from exponential phase cultures (OD_600_ = 0.8) by the SDS-boiling procedure as described for *Streptococcus pneumoniae* (Bui et al., 2012). Purified cell wall samples were lyophilized and used for analysis of glycosyl composition.

### Glycosyl composition analysis

Carbohydrate composition analysis was performed at the Complex Carbohydrate Research Center (Athens, GA).

#### Mild hydrolytic conditions

Glycosyl composition analysis was performed by combined gas chromatography—mass spectrometry (GC-MS) of the per-O-trimethylsilyl (TMS) derivatives of the monosaccharide methyl glycosides produced from the sample by acidic methanolysis as described previously by Santander et al. (2013). Briefly, the sample (500 μg) with myo-inositol (internal standard, 20 μg) was heated with methanolic HCl in a sealed screw-top glass test tube for 18 h at 80°C. After cooling and removal of the solvent under a stream of nitrogen, the samples were treated with a mixture of methanol, pyridine, and acetic anhydride for 30 min. The solvents were evaporated, and the samples were derivatized with Tri-Sil® (Pierce) at 80°C for 30 min. GC-MS analysis of the TMS methyl glycosides was performed on an Agilent 7890A GC interfaced to a 5975C MSD, using an Supelco Equity-1 fused silica capillary column (30 m × 0.25 mm ID).

#### Strong hydrolytic conditions

Samples were subjected to a strong aqueous hydrolytic step, prior to performing the methanolysis procedure described above. For strong hydrolysis, the dried samples, including internal standard myo-inositol, were hydrolyzed in 4 M HCl for 3 h at 105°C. The HCl was removed under nitrogen stream, and the residue was treated with water and re-evaporated using a Speed-Vac rotary vacuum evaporator. Traces of water were then removed by evaporating with anhydrous methanol in the Speed–Vac. The dried samples were then subjected to methanolysis, N-acetylation, and trimethylsilylation as described above.

### Statistical analysis

Unless otherwise indicated, statistical analysis was carried out from at least three independent experiments. Quantitative data were analyzed using the paired Student's *t*-test. A *P*-value equal to or less that 0.05 is considered statistically significant.

## Results

### Phenotypic characterization of *spyB* and *spyA* deletion mutants

In order to identify SpyB function we generated an in-frame deletion in *spyB* of 51 bp (deletion of codons 9–25) in the hyperinvasive M1T1 serotype strain, MGAS5005 (Sumby et al., 2005), creating the 5005Δ*spyB* mutant (Figure S1). As we previously reported (Korotkova et al., 2012) this deletion did not have an effect on SpyA translational fusion using a plasmid-based reporter system. Moreover 5005Δ*spyB* expressed equivalent levels of *spyA* (Figure S2A) confirming that the deletion has no a polar effect on gene expression levels. The SpyA deficient mutant, 5005Δ*spyA*, was previously constructed in the same strain background (Hoff et al., 2011) and utilized for our experiments. Whole-genome sequencing was performed for both mutants to confirm the deletion and the absence of additional mutations.

**Figure 1:**
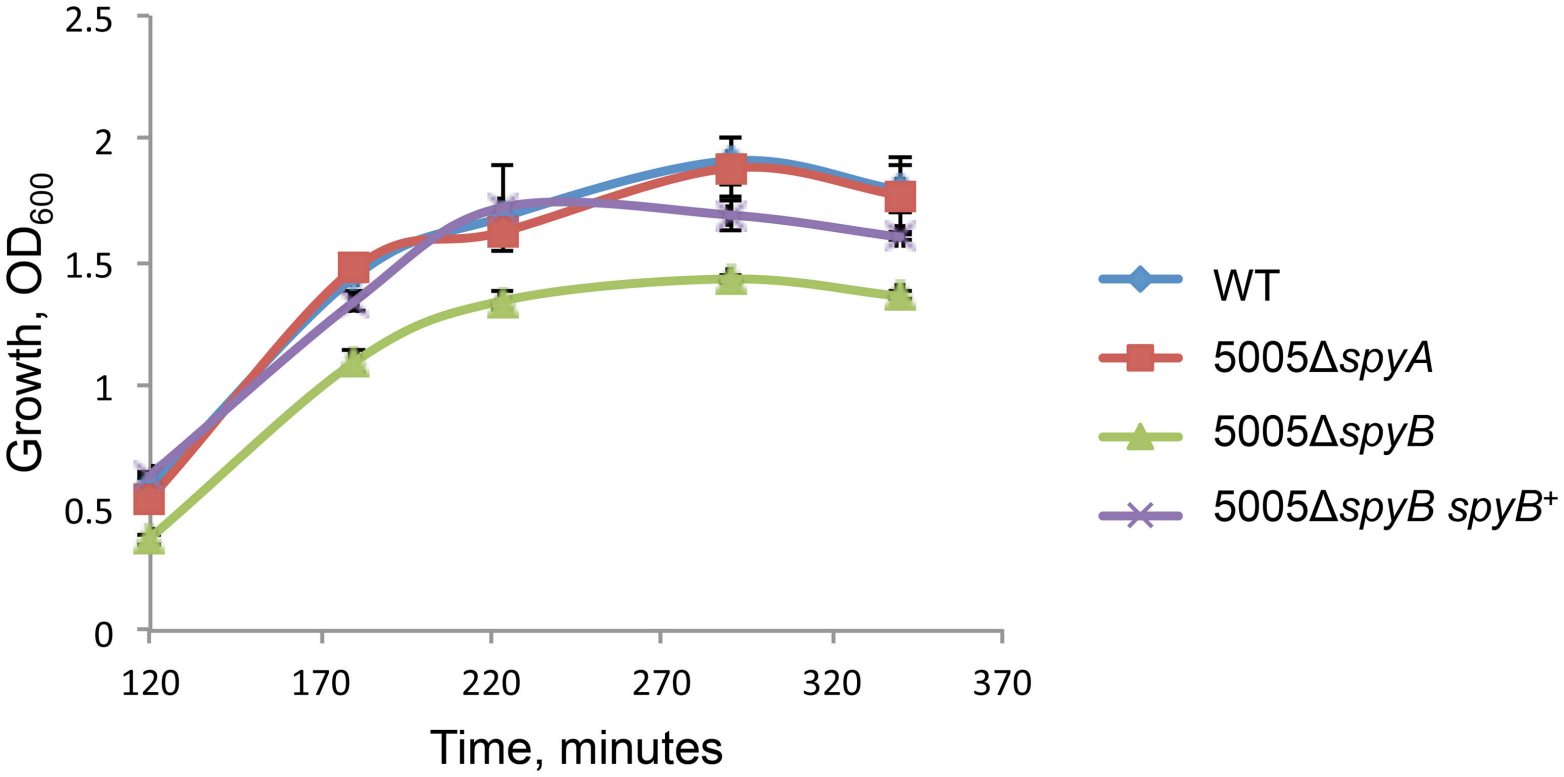
**Growth of the MGAS5005 (WT), 5005Δ*spyB*, 5005Δ*spyB spyB*^+^, and 5005Δ*spyA* in THY medium**. The data are the mean of three independent experiments ± standard deviation.

**Figure 2:**
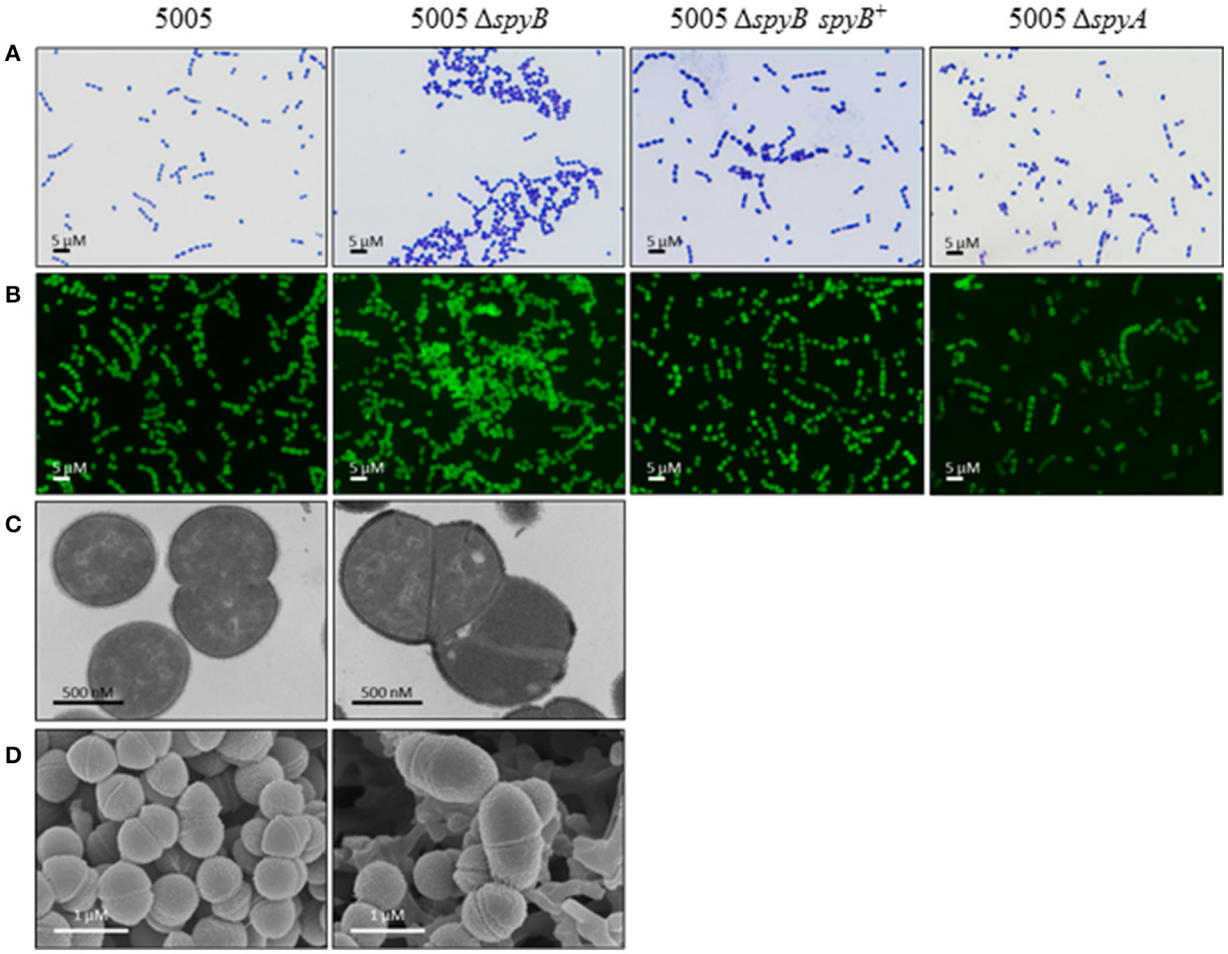
**SpyB deficiency causes MGAS5005 cells to aggregate**. MGAS5005, 5005Δ*spyB*, 5005Δ*spyB spyB*^+^, and 5005Δ*spyA* strains at middle exponential phase were examined by microscopy. **(A)** MGAS5005 strains were Gram stained and imaged by light microscopy. **(B)** MGAS5005 strains were stained with fluorescently labeled vancomycin to visualize the cell walls and septa, and imaged by fluorescence microscopy. **(C)** Fixed cultures of MGAS5005 and 5005Δ*spyB* were visualized by transmission electron microscopy or **(D)** scanning electron microscopy.

5005Δ*spyB* demonstrated impairment in growth in nutrient-rich THY medium (Figure 1). Gram staining of bacteria revealed that the exponentially grown *spyB* deletion mutant forms large cellular aggregates instead of typical chains of ovococci formed by the WT strain and 5005Δ*spyA* (Figure 2A). By complementing the 5005Δ*spyB* mutant *in cis* with *spyB* (5005Δ*spyB spyB*^+^), we restored the WT phenotype (Figure 2A). This observation indicated that the *spyB* mutant is defective in cell division or cell separation. Fluorescent microscopy of bacteria labeled with a fluorescent derivative of a cell wall and septa-binding antibiotic, vancomycin, indicated no obvious defects in cell division for 5005Δ*spyB* (Figure 2B). Although some instances of improper septa formation were visible by transmission and scanning electron microscopy with comparison to WT (Figures 2C,D). These data suggest that the increased aggregation is most likely due to defective cell separation.

**Figure 3:**
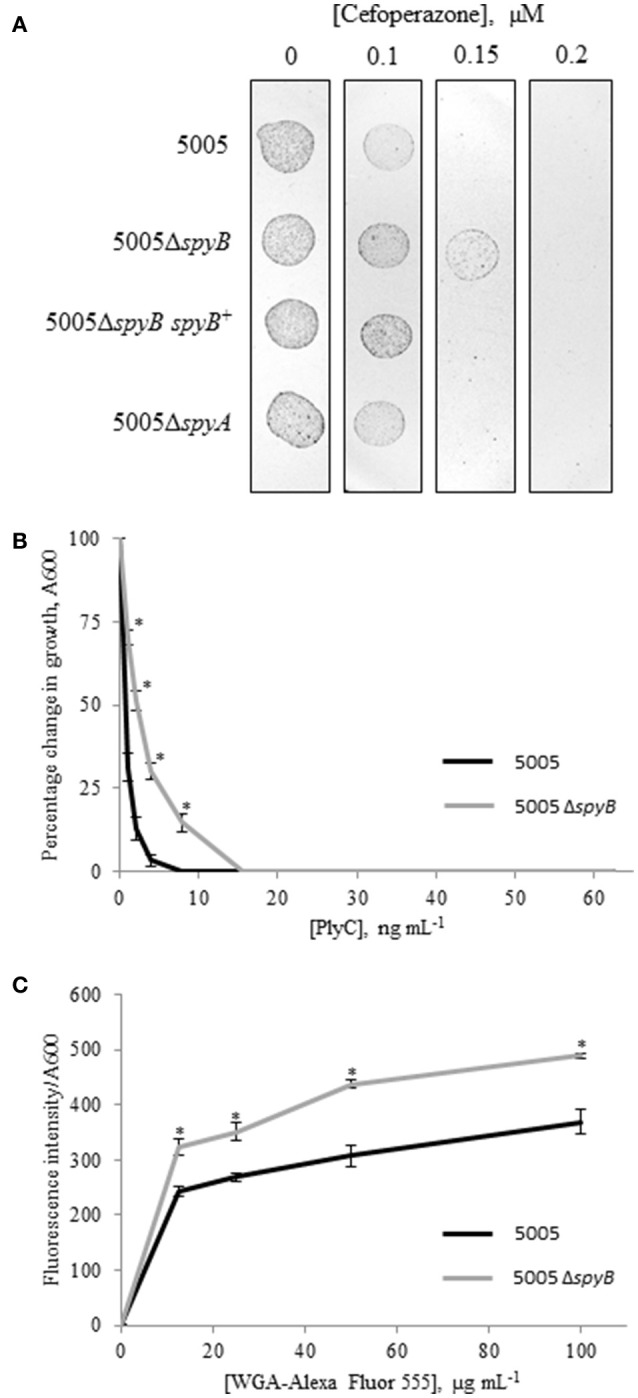
**Phenotypic analysis of 5005Δ*spyB*. (A)** SpyB deficiency promotes resistance to cefoperazone. MGAS5005 strains were grown in CDM to middle exponential phase, diluted in PBS (10^−2^ dilution) and then plated on THY agar supplied with 0, 0.1, 0.15, and 0.2 μM cefoperazone in triplicate. This result is representative of five independent experiments (*P* = 0, *t*-test). **(B)** SpyB deficiency decreases the sensitivity of MGAS5005 to the PG hydrolase PlyC. MGAS5005 and 5005Δ*spyB* at middle exponential phase were measured for growth at 600 nm following 60 min incubation in the presence of various concentrations of PlyC (0–62.5 ng mL^−1^). The average percentage change in growth ± standard deviation for three replicates is shown, the asterisk indicates values that are statistically different (*p* < 0.01) from WT 5005. **(C)** SpyB deficiency alters the affinity of MGAS5005 for GlcNA binding wheat germ agglutinin (WGA). MGAS5005 and 5005Δ*spyB* at middle exponential phase were incubated for 60 min with various concentrations (0–100 μg mL^−1^) of WGA conjugated to Alexa Fluor 555. After washing the bacteria were measured for fluorescence (ex 544 nm, em 590 nm) and normalized for growth at 600 nm. Data are the average of three replicates ± standard deviation, the asterisk indicates values that are statistically different (*p* < 0.01) from WT 5005.

To identify additional phenotypes of the mutants, we utilized Biolog phenotype MicroArrays that allow quantitative screening of growth and metabolic activities of the WT, 5005Δ*spyB*, and 5005Δ*spyA* mutant strains under various metabolic conditions. The latter included various carbon, nitrogen, phosphate, and sulfur sources; osmolytes; metabolic inhibitors; and toxic compounds including several types of antibiotics. The analysis revealed stronger growth of 5005Δ*spyB* in comparison to WT and 5005Δ*spyA* in the presence of β-lactam antibiotics of the cephalosporin class: cefoperazone, cefamandole nafate, cefsulodin, and cefoxitin (Figure S3). The cellular targets of cephalosporins are penicillin-binding proteins (PBPs) that catalyze the final steps of peptidoglycan (PG) biosynthesis. The antibiotics block the formation of peptide cross-links in the PG catalyzed by the transpeptidase domain of PBPs. To verify the increased cephalosporin resistance of 5005Δ*spyB*, strains were grown in nutrient-limited CDM to middle exponential phase and then plated on THY agar supplied with different concentrations of cefoperazone. We found that the minimum inhibitory concentration (MIC) of cefoperazone for the WT, 5005Δ*spyA*, and 5005Δ*spyB spyB*^+^ strains was 0.15 μM (Figure 3A). 5005Δ*spyB* showed decreased susceptibility to the antibiotic with a MIC of 0.2 μM. Although the MIC-values varied by less than two-fold, differences between the strains were highly reproducible (*P* = 0, *t*-test; *n* = 5).

Bacteria have several PBPs that display variabilities in binding profiles for different β-lactams resulting in selective antimicrobial effects (Hakenbeck et al., 2012). The β-lactams include antibiotics of the penicillin class such as penicillin G, methicillin, and ampicillin. To determine whether SpyB deficiency confers resistance to the penicillin class of β-lactams, we analyzed the sensitivity of the strains to methicillin and ampicillin. We found no significant differences in penicillin antibiotic susceptibility between strains.

### SpyB does not affect the abundance of PBPs in the cell membrane

In contrast to enterococci and staphylococci, GAS does not produce β-lactamases, which cleave the β-lactam group of antibiotics. In many instances the mechanisms of resistance to β-lactam antibiotics are due to amino acid substitutions in PBPs, leading to altered activity or stability of PBPs (Albarracin Orio et al., 2011). GAS genomes encode four HMW PBPs that are homologs of *S. pneumoniae* PBPs —three are class A PBPs (PBP1A, 1B, and 2A) and one is a class B PBP, PBP2X (Philippe et al., 2014; Table S4). Furthermore, HMW PBPs might be present in the cell membrane in several molecular forms due to the presence of alternate start codons that are preceded by Shine-Dalgarno sequences. Additionally, we identified three low-molecular-weight (LMW) class C PBPs in the GAS genomes.

**Figure 4:**
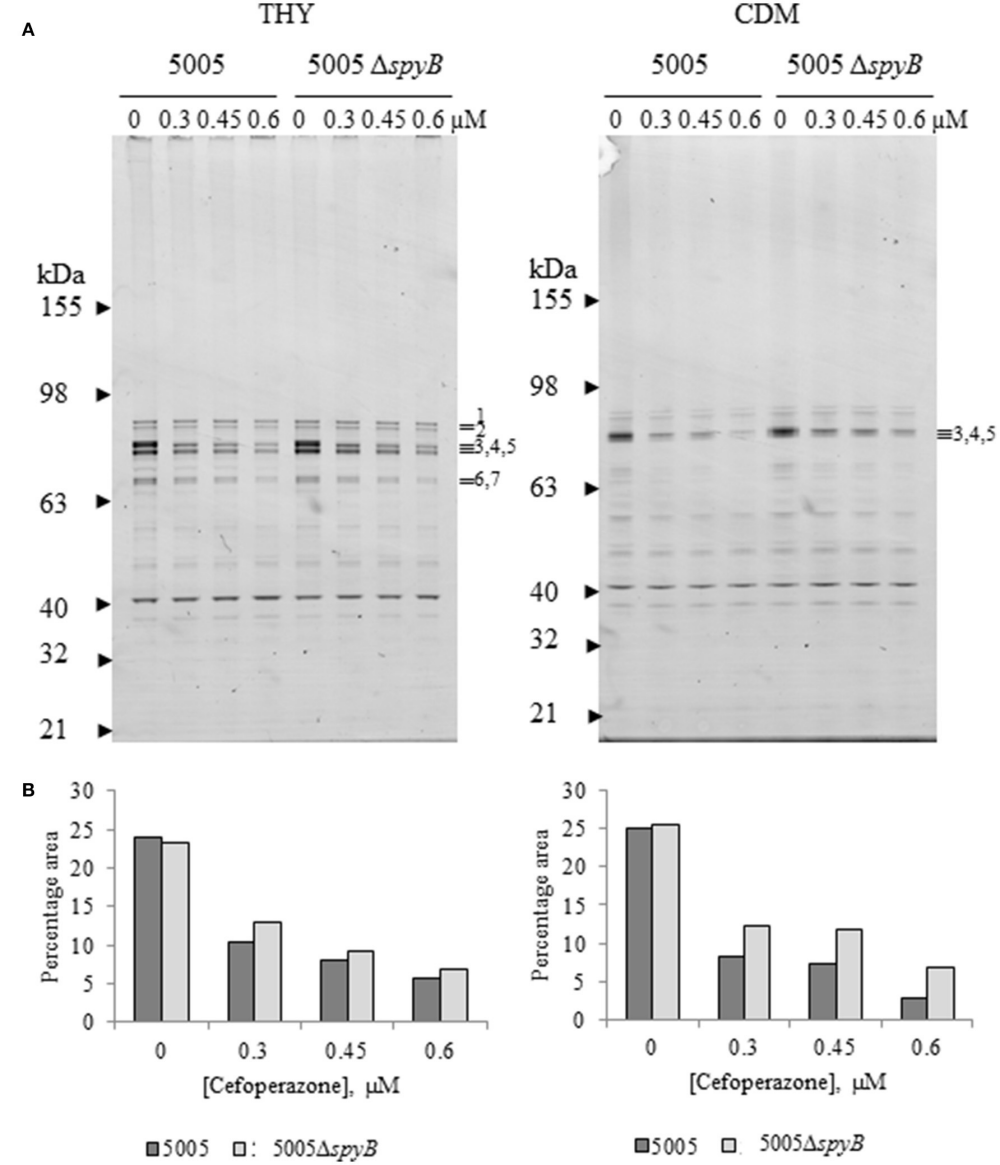
**Cefoperazone inhibition of PBPs in MGAS5005 and 5005Δ*spyB*. (A)** Gel-based analysis of PBP expression in MGAS5005 strains grown in THY medium or CDM. Whole cells were treated with various concentrations of cefoperazone and subsequently labeled with Boc-FL. Membrane fractions were separated on 4–12% SDS-PAGE gels and visualized as described in Materials and Methods. **(B)** Densitometry analysis of bands 3, 4, and 5 for the gels shown in panel **(A)**. Data are representative of biological triplicates.

To investigate a putative role of SpyB in the regulation of PBPs, we set out to characterize the effect of cefoperazone on the abundance of PBPs in MGAS5005 and 5005Δ*spyB*. PBPs can be visualized using fluorescent β-lactams that form a covalent complex with PBPs (Kocaoglu et al., 2012). Strains were grown in THY broth or CDM, and exponentially growing cultures were treated with different concentrations of cefoperazone followed by incubation with an excess of Bocillin FL (Boc-FL), fluorescent penicillin. Gel-based analysis of THY-grown GAS identified seven major protein bands with molecular weights (MW) ranging between 63 and 98 kDa, and one major band with a MW of ~40 kDa (Figure 4A). The MW of the major protein bands correlated with the sizes of HMW and LMW PBPs encoded by the GAS genome (Table S4). Additionally, Boc-FL-treated bacteria displayed multiple minor bands (Figure 4A). Growth in CDM reduced the number of visible PBPs (Figure 4A). Treatment with an increasing concentration of cefoperazone resulted in a decrease in these HMW bands in THY and CDM-grown bacteria. Band 3 was the first to disappear from the gel indicating that the corresponding PBP has the strongest affinity for cefoperazone. Densitometry analysis of the bands revealed no significant differences in Boc-FL labeling in the MGAS5005 in comparison to 5005Δ*spyB* (Figure 4B). Thus, this analysis suggested that SpyB does not affect the abundance of PBPs in the cell membrane.

### The composition of cell wall is altered in the *spyB* mutant

GAS cell wall is composed of PG and the covalently attached rhamnose (Rha)-containing group A-specific carbohydrate (GAC; Mistou et al., 2016). Fluorescent microscopy of the PG-binding antibiotic vancomycin indicated that 5005Δ*spyB* was not defective in PG formation (Figure 2B). However, we found that the mutant has decreased sensitivity to bacteriophage-encoded PG hydrolase PlyC (Figure 3B), whereas the sensitivity to PG hydrolase mutanolysin was not affected (data not shown). PlyC, is known to lyse streptococcal species bearing a polyrhamnose epitope (Nelson et al., 2001), indicating that similar to other bacteriophage PG hydrolases, PlyC recognizes a carbohydrate epitope in the cell wall (Nelson et al., 2006). Structural studies of GAC have shown that the polysaccharide consists of alternating α(1,2)- and α(1,3)-linked Rha units and side units of β-N-acetylglucosamine (GlcNA) linked to the O-3 position of the polyrhamnose backbone (Coligan et al., 1978; Huang et al., 1986). Given the changes in sensitivity to PlyC of 5005Δ*spyB*, we evaluated the ability of bacteria to bind to Alexa Fluor 555-labeled WGA, a lectin that specifically binds GlcNA. Deletion of *spyB* led to increased binding of WGA compared with the WT strain (Figure 3C). This suggests that the mutant has increased amounts of GlcNA-containing saccharides on the cell surface.

In order to investigate whether GAC production is altered in the *spyB* deletion mutant, we analyzed the concentration of total carbohydrates in bacteria. The results of total carbohydrate analysis showed that there was no difference between MGAS5005 and 5005Δ*spyB* (Table S5).

To determine whether the monosaccharide composition of GAC is modified in 5005Δ*spyB*, we performed glycosyl composition analysis on cell wall material isolated from MGAS5005 and 5005Δ*spyB*. The analysis was carried out on two independent biological replicates. The Rha/GlcNa/N-acetylmuramic acid (MurNA) content of isolated cell wall fractions was determined by GC-MS analysis of the trimethylsilyl methyl-glycosides following methanolysis, mild hydrolysis, (0.8 N methanolic HCl, 80°C, 16 h) and re-N-acetylation of amino sugars (Chambers and Clamp, 1971). The second analysis (strong hydrolysis) included a strong acid pre-treatment (4 M HCl, 105°C, 3 h) to promote better hydrolysis of 2-amino sugars. The strong acid pretreatment improves recovery of monosaccharides linked to 2-amino sugars, but it is important to bear in mind that certain labile sugars, such as Rha, may be partially destroyed under these conditions. Mild cleavage conditions promoted the release of only 3–28% w/w carbohydrates from MGAS5005 cell wall (Table 1, Table S6 and Figure S4). The same hydrolytic conditions released 47–49% w/w carbohydrates from 5005Δ*spyB* cell wall (Table 1, Table S6 and Figure S4). The use of strong hydrolytic conditions promoted a significant increase in Rha, GlcNA and MurNA recoveries from MGAS5005 cell wall (Table 1, Table S6 and Figure S4), with yields of 64–92%. In contrast, strong hydrolysis was as affective as methanolysis in recovery of the carbohydrates from 5005Δ*spyB* cell wall (Table 1, Table S6 and Figure S4), with yields of 32–45%. This observation could indicate that the WT cell wall contains an increase in GlcNA side-chains, that are resistant to hydrolysis. The strong acid treatment would promote cleavage of the –GlcNA–Rha glycosidic linkage, yielding larger amounts of Rha and GlcNA in the WT strain compared with mild hydrolysis. Alternatively, there could be differences in de-acetylation of GlcNA because cleavage of the bond between de-acetylated GlcNA (GlcN) and Rha would require stronger acidic conditions than hydrolysis of the bond between neutral sugars GlcNA and Rha (Merkle and Poppe, 1994; Jennings et al., 2015). De-acetylation of GlcNA would be undetectable due to the N-acetylation step prior to GC-MS. Our binding experiments with GlcNA-specific lectin WGA supported the latter explanation. In conclusion our analyses indicated that there are differences in the cell wall composition of 5005Δ*spyB* compared with MGAS5005.

**Table 1:** **Glycosyl composition analysis of MGAS5005 and 5005Δ*spyB* cell wall**.

	**Residue**	**Mild hydrolysis**			**Strong hydrolysis**		
		**Weight (μg CHO^a^/100 μg)**	**nmol (nmol CHO/100 μg)**	**mol (%)**	**Weight (μg CHO/100 μg)**	**nmol (nmol CHO/100 μg)**	**mol (%)**
MGAS5005	Rha	2.2	134.4	86.5	39.8	2425.8	50.6
	GlcNA	0.5	21.0	13.5	51.5	2330.1	48.6
	MurNA	0.0	0.0	0.0	1.0	34.0	0.7
5005Δ*spyB*	Rha	28.3	1726.2	67.7	24.3	1480.7	61.4
	GlcNA	18.1	819.0	32.1	20.4	920.8	38.2
	MurNA	0.2	5.6	0.2	0.3	10.4	0.4

a *CHO, carbohydrate*.

### SpyB binds heme and protoporphyrin IX

A major problem in studying SpyB function *in vitro* is the small size of this protein, making it challenging to produce recombinant protein for thorough biochemical analysis. To circumvent this problem, a fusion of maltose binding protein (MBP) to SpyB was constructed. We observed that MBP-SpyB was purified as a brown colored protein following its expression in *Escherichia coli* and purification by Ni-NTA affinity chromatography (Figures S5A,B). When expressed alone, purified MBP was not found in a colored form, implying that the chromophore associates with SpyB. We suggest that the MBP-SpyB is brown-colored because of the interaction between the protein and the chromophore, heme. To test this hypothesis we separated MBP-SpyB on a native PAGE gel followed by staining with tetramethylbenzidine, a chromogenic compound that changes color in the presence of heme-associated peroxidase activity. This activity was localized to MBP-SpyB, and it was absent from the MBP only control lane (Figure S5C).

**Figure 5:**
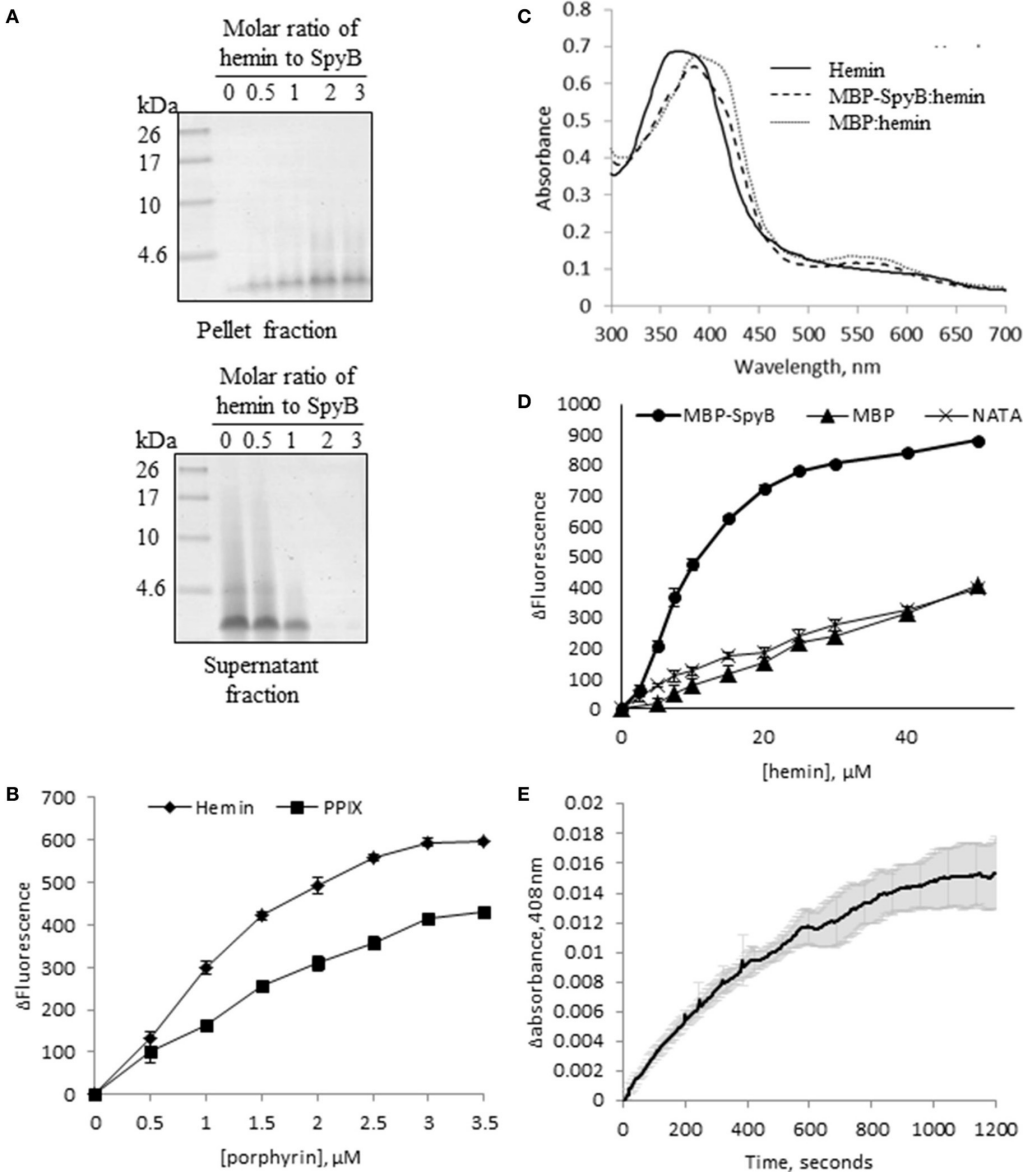
**SpyB is a porphyrin-binding protein. (A)** Hemin binding decreases SpyB solubility. SpyB was mixed with hemin in different molar ratios. Soluble and insoluble fractions were separated by centrifugation and resolved on a 16% Tris-Tricine gel. **(B)** The change in tryptophan fluorescence quenching of SpyB by hemin and protoporphyrin IX (PPIX). A series of hemin or PPIX concentrations (0, 0.5, 1, 1.5, 2, 2.5, 3, and 3.5 μM) was added to 5 μM SpyB, in the presence of 1 mM DTT. The curves are the average of three replicates ± standard deviation. The dissociation constant (K_D_) was calculated using SigmaPlot software. **(C)** Absorbance spectra between 300 and 700 nm were collected for 50 μM hemin; 50 μM hemin mixed with 2.5 μM MBP-SpyB; and 50 μM hemin mixed with 2.5 μM MBP, in the presence of 1 mM DTT. **(D)** The change in tryptophan fluorescence quenching for MBP-SpyB and MBP. A series of hemin concentrations (0, 2.5, 5, 7.5, 10, 15, 20, 25, 30, 40, 50 μM) was added to 2.5 μM MBP-SpyB monomer or MBP, in the presence of 1 mM DTT. A control experiment was performed with 27.5 μM N-acetyltryptophanamide (NATA) under the same conditions. The curves are the average of three replicates ± standard deviation. The K_D_ was calculated using SigmaPlot software. **(E)** A time course for hemin transfer from MBP-SpyB to apomyoglobin was measured at 408 nm over 20 min. The dissociation rate constant (K_off_) was calculated from the change in absorbance at 408 nm using Graphpad Prisim software. Data are the average of three replicates ± standard deviation. The K_off_ along with the previously measured K_D_ were used to calculate the binding rate constant (K_on_) for MBP-SpyB.

The term heme refers to reduced, ferrous or Fe (II) iron protoporphyrin IX, whereas the term hemin refers to the oxidized, ferric or Fe(III) form of the molecule. *E. coli* displays a complete heme biosynthetic pathway, predominantly producing the ferrous form. We attempted to confirm that heme is associated with SpyB by analyzing hemin binding to synthetic SpyB peptide. The peptide was water-soluble, however the addition of hemin to SpyB significantly changed the protein solubility resulting in the precipitation of SpyB in aqueous solution (Figure 5A). Furthermore, analysis of the synthetic peptide mixed with hemin by size-exclusion chromatography was hampered by a strong non-specific interaction between SpyB and the resin, when we utilized a Superdex Peptide 10/300 GL (GE Healthcare Life Sciences) column. We were however, able to determine the affinity using tryptophan fluorescence quenching (Shepherd et al., 2007). As shown in Figure 5B (diamonds), hemin quenched tryptophan fluorescence of the synthetic SpyB in a dose-dependent manner. The resulting saturation curve allowed us to estimate a K_D_-value of 2.6 × 10^−6^ ± 0.6 × 10^−6^ M. Next, we investigated whether other porphyrins, such as protoporphyrin IX (the iron-free precursor of heme) and vitamin B_12_, bind SpyB. As shown in Figure 5B (squares), SpyB interacted with protoporphyrin IX with a K_D_-value of 4.9 × 10^−6^ ± 0.8 × 10^−6^ M. In contrast, vitamin B_12_ did not modulate the intrinsic fluorescence of SpyB, indicating that there is no interaction (data not shown).

Further investigations into hemin binding utilized the MBP-SpyB construct. Firstly, we obtained pure MBP-SpyB for use in recording the absorption spectra in the presence of hemin. Because SpyB is a cysteine-rich protein, dithiothreitol (DTT) was included in the buffers used for size-exclusion chromatography and for heme-binding assays. DTT helps in preventing formation of non-specific intramolecular disulfide linkages. We found that DTT-treated MBP-SpyB preparations obtained from recombinant *E. coli* were eluted in two peaks following size-exclusion chromatography, with the majority eluting in the lower molecular weight peak, which we predicted to be monomeric MBP-SpyB (Figure S6). Further analysis of the protein present in the small, high molecular weight peak identified MBP-SpyB associated with heme. Therefore, to demonstrate hemin binding by MBP-SpyB, we used the protein from the low molecular weight, heme-free peak. The absorption spectrum of hemin at pH 7.5 exhibited a broad peak between 355 and 400 nm (Figure 5C, solid line). When MPB-SpyB was added to the hemin solution, a double peak was observed with absorption maxima at 360 and 385 nm (dashed line). The addition of MBP shifted the peak but did not alter the shape of the hemin spectrum (Figure 5C, dotted line). The affinity of the hemin/MBP-SpyB interactions was determined by quenching of intrinsic tryptophan fluorescence (Shepherd et al., 2007). This method estimated that MBP-SpyB binds hemin with a K_D_-value of 1.2 × 10^−5^ ± 0.4 × 10^−5^ M (Figure 5D, circles). The addition of hemin did not quench the fluorescence of MBP (Figure 5D, triangles), compared with the control N-acetyltryptophanamide (NATA) (crosses), which further demonstrates that MBP alone does not bind hemin. In addition, the Stern-Volmer plots for these data (Figure S7) showed weak dynamic quenching of MBP and NATA by hemin under our experimental conditions.

To gain insights into the mechanism of hemin binding to SpyB, we measured the dissociation rate constant using apomyoglobin as a hemin scavenger (Figure 5E). The hemin dissociation rate constant K_off_ for MBP-SpyB reconstituted with hemin was 1.8 × 10^−3^ ± 0.07 × 10^−3^ s^−1^. The associate rate constant K_on_ = 1.5 × 10^2^ ± 0.3 × 10^2^ M^−1^ s^−1^ was calculated using the equilibrium constant K_D_ for MBP-SpyB. The data indicated that although hemin forms a stable complex with MBP-SpyB, it is not bound tightly. The K_D_-value of hemin binding by MBP-SpyB is lower than the reported affinities of heme-binding proteins involved in heme scavenging from host (Ortiz de Orué Lucana et al., 2014). However, the K_D_-value is within the range reported for some bacterial heme-sensing proteins, such as a regulator of porphyrin efflux system, PefR (Sachla et al., 2014), a repressor of photosystem genes, PpsR (Yin et al., 2012), light-regulated antirepressor, AppA (Yin et al., 2013), and a redox stress regulator, HbpS (Ortiz de Orué Lucana et al., 2014). Thus, these results suggest that SpyB plays a role in binding heme and possibly serves as an environmental sensor in GAS.

### Heme binding induces SpyB dimerization

**Figure 6:**
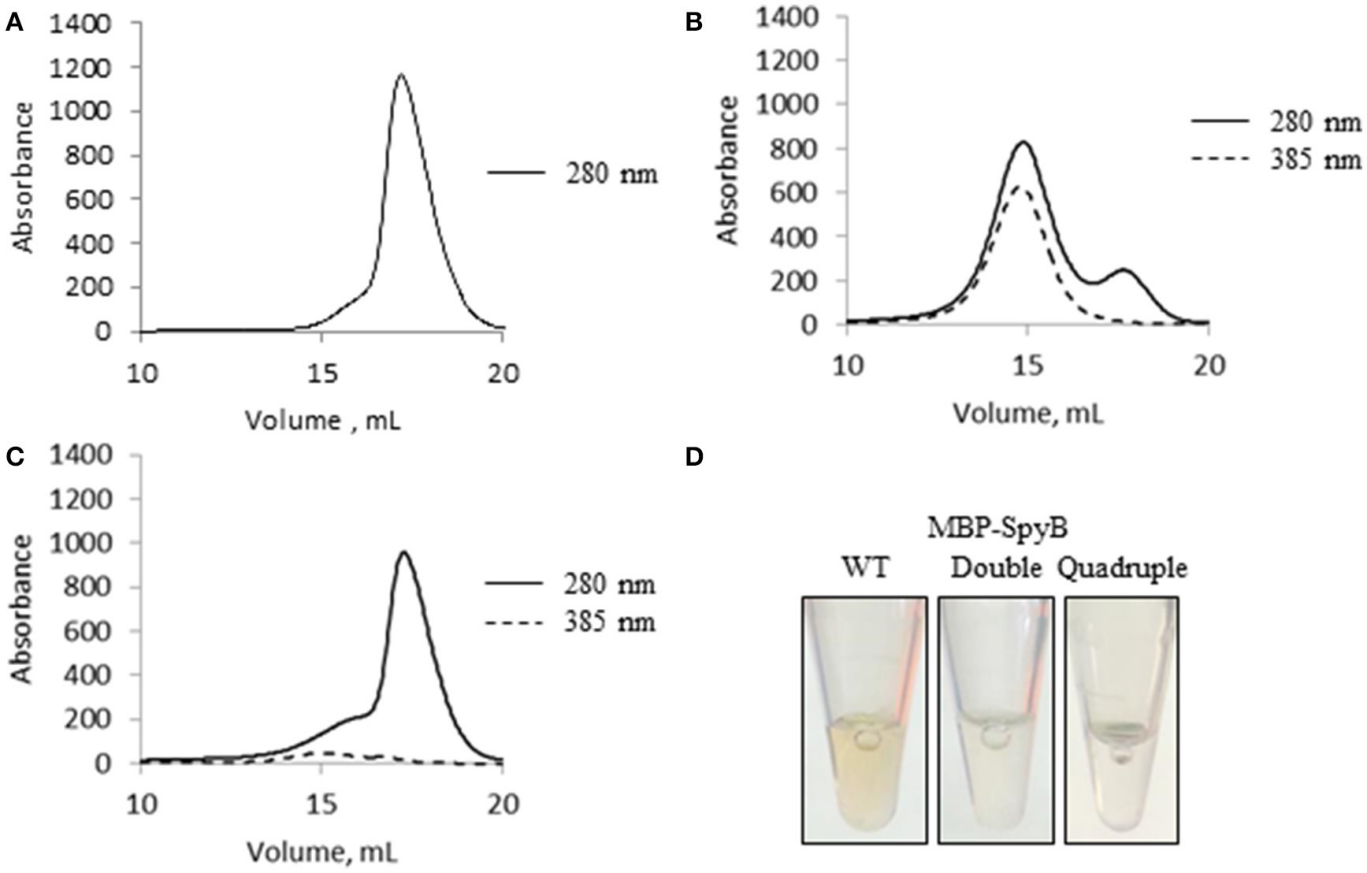
**Hemin binding induces SpyB oligomerization. (A)** The size-exclusion chromatogram of MBP-SpyB monomer. **(B)** The size-exclusion chromatogram of MBP-SpyB monomer reconstituted with hemin in 1:4 ratio. **(C)** The size-exclusion chromatogram of MBP-SpyB monomer reconstituted with protoporphyrin IX in 1:4 ratio. The absorbance was monitored at 280 and 385 nm (hemin and protoporphyrin IX detection). **(D)** The appearance of WT MBP-SpyB, and double (C7A/C13A) and quadruple (C7A/C13A/C30A/C35A) MBP-SpyB mutants, following expression in *E. coli* Rosetta DE3 cells and purification by Ni-NTA affinity chromatography and size-exclusion chromatography. Data are representative of biological triplicates.

The significant change in synthetic SpyB peptide solubility and the chromatogram for MBP-SpyB suggested that hemin induces structural changes, likely dimerization, in SpyB. To investigate whether hemin binding affects the multimeric state of SpyB, we analyzed the purified heme-free MBP-SpyB (Figure 6A) mixed with hemin in a 1:4 ratio by size-exclusion chromatography. The size-exclusion chromatogram revealed two distinct peaks (Figure 6B). The absorbance at 385 nm, for detection of hemin, aligned only with the high molecular weight peak, and the protein that eluted during this peak had a brown color (Figure 6D). Dynamic light scattering analysis of the main fraction collected during elution of the high molecular weight peak confirmed that MBP-SpyB was in a dimeric state (Figure S8). To calculate the amount of hemin bound to MBP-SpyB dimer, we employed the pyridine hemochrome assay. We found that the ratio of MBP-SpyB:hemin was two per dimer (Figure S9). The calculated extinction coefficient of MBP-SpyB:hemin was 31405 M^−1^ cm^−1^.

Additionally, we analyzed whether protoporphyrin IX binding to SpyB induces protein dimerization. In contrast to hemin, reconstitution of MBP-SpyB with protoporphyrin IX did not significantly change the monomeric state of MBP-SpyB (Figure 6C), and the protoporphyrin IX largely dissociated from MBP-SpyB. Taken together, our data demonstrated that the association of iron-chelated porphyrin with SpyB induces MBP-SpyB dimerization.

### SpyB cysteine residues are involved in heme binding and dimerization

**Figure 7:**
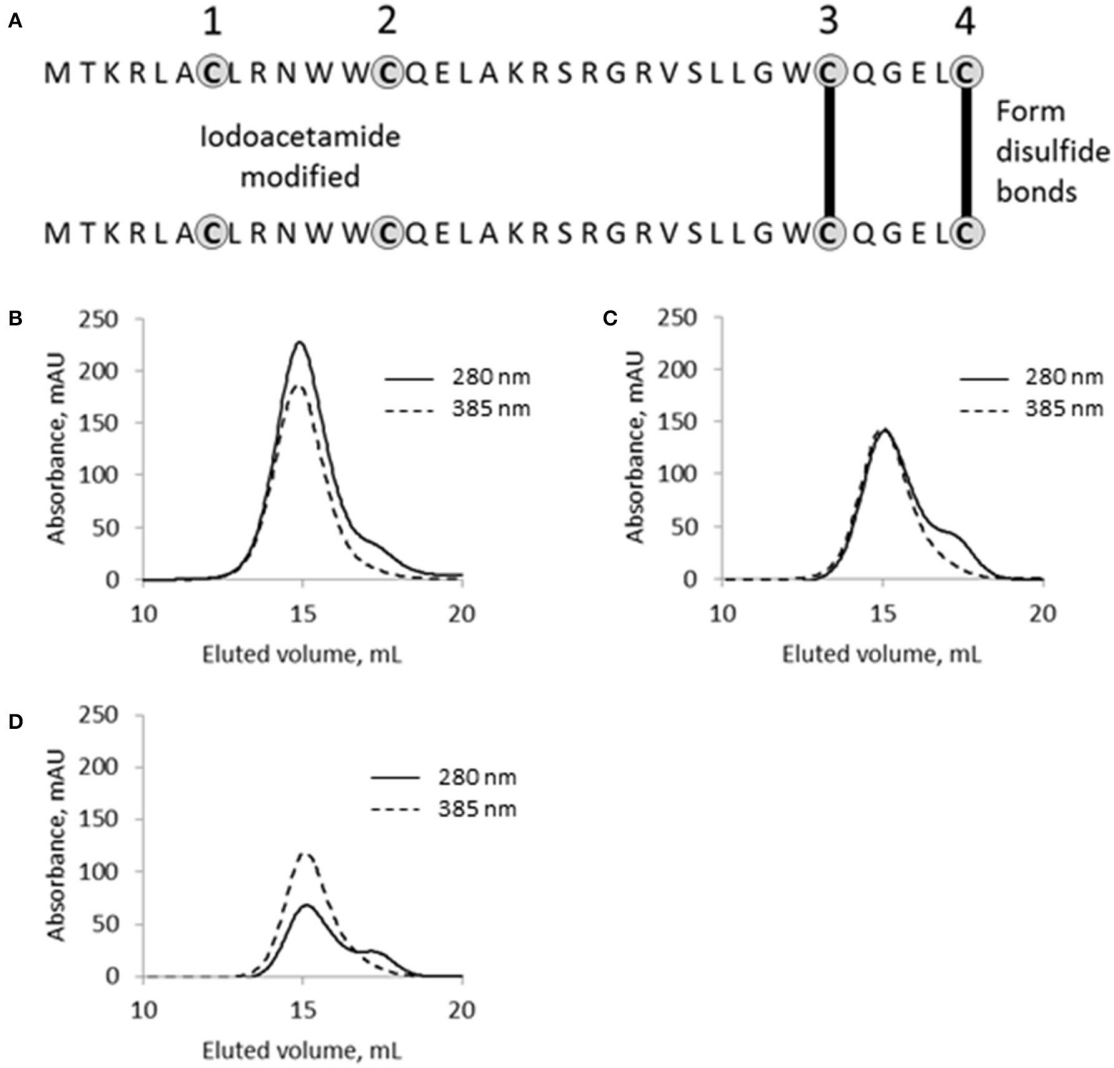
**The functional role of cysteine residues in SpyB. (A)** Schematic of SpyB modification by iodoacetamide. The size-exclusion chromatogram of **(B)** MBP-SpyB WT, **(C)** double (C7A/C13A), and **(D)** quadruple (C7A/C13A/C30A/C35A) mutants reconstituted with hemin. The absorbance was monitored at 280 and 385 nm (hemin). Data are representative of biological triplicates.

SpyB contains four cysteines, which indicates their functional significance given the small size of the protein. Cysteine residues can form a covalent bond with the vinyl groups of heme (Bowman and Bren, 2008). They can also participate in non-covalent heme binding and/or disulfide bond formation. To examine the role of the cysteine residues in heme binding and dimerization we employed iodoacetamide (IAA) treatment followed by mass spectrometry (MS) analysis. IAA treatment causes the addition of a carbamidomethyl group to free cysteines. Using this method, we detected that treatment of MBP-SpyB dimer with DTT followed by incubation with IAA resulted in the modification of all four cysteines. In contrast, treatment of MBP-SpyB dimer with IAA alone generated a protein with the first and second cysteine residues modified by IAA. The MS analysis identified two intermolecular disulfide bonds involving the third and fourth cysteines (Figure 7A).

From this experiment, we concluded that SpyB cysteine residues do not participate in the covalent binding of heme. To test whether the first and second cysteines are involved in non-covalent binding of heme, we generated double (C7A/C13A) and quadruple (C7A/C13A/C30A/C35A) mutant proteins. We noticed that only the quadruple mutant protein was colorless following expression in *E. coli* and purification by Ni-NTA affinity chromatography and size-exclusion chromatography (Figure 6D). In addition, the quadruple mutant protein was also eluted from the size-exclusion column as a single peak corresponding to a monomeric state of SpyB (data not shown). The double mutant protein had a pale brown color after the two purification steps (Figure 6D) and eluted from the size-exclusion column as a dimer and monomer peak (data not shown). The mutant proteins were reconstituted with hemin as described in Materials and Methods Section and analyzed by size-exclusion chromatography. As shown in Figures 7B–D, hemin induced dimerization of both mutant variants. However, the removal of all the cysteines resulted in an elution profile displaying a major monomer peak and a significantly smaller dimer peak. Analysis of hemin content in the dimers of the mutant SpyB proteins by pyridine hemochrome assay displayed no changes in the MBP-SpyB/hemin ratio (Figure S9). Tryptophan fluorescence quenching determination of hemin binding affinities for the mutant variants revealed that the removal of cysteines led to a significant decrease in affinity for hemin (Table 2). The Stern-Volmer plots (Figure S7) further demonstrated the decrease in quenching capacity of hemin for the double and quadruple mutants compared with WT. Taken together, our data demonstrated that SpyB cysteine residues are involved in disulfide bond formation between SpyB subunits and they contribute to non-covalent heme association with the protein.

**Table 2:** **Hemin and protoporphyrin IX binding constants for SpyB protein variants**.

**Protein**	**Hemin, K_D_ (M)**	**Protoporphyrin IX, K_D_ (M)**
SpyB	2.6 × 10^−6^ ± 0.6 × 10^−6^	4.9 × 10^−6^ ± 0.8 × 10^−6^
MBP-SpyB	1.2 × 10^−5^ ± 0.4 × 10^−5^	ND^a^
MBP-SpyB C7A/C13A mutant	3.7 × 10^−5^ ± 1.2 × 10^−5^	ND
MBP-SpyB C7A/C13A/C30A/C35A mutant	3.2 × 10^−4^ ± 2.1 × 10^−4^	ND

a *Not determined*.

## Discussion

Small proteins of 50 and fewer amino acids are a relatively unexplored territory in biological research. The proteins encoded by ORFs smaller than 100 codons are typically ignored during genome annotations. Moreover, functional characterization of small proteins presents significant challenges. Mutations of genes encoding the majority of small proteins identified thus far have no strong phenotypes because typically small proteins function as modulators of the activities of larger proteins with which they interact. Nevertheless, emerging data illustrate the importance of small proteins in biological processes (Storz et al., 2014). Curiously, the majority of small bacterial proteins identified thus far are located in membranes (Storz et al., 2014).

In this report, we undertook biochemical and functional characterization of a 35-amino acid protein, SpyB, present in the human pathogen, *S. pyogenes*. Besides its small size, the protein possesses fascinating structural and functional characteristics including an N-terminal amphipathic helix, a number of conserved cysteine residues, membrane localization, and a functional link with the membrane-associated ADP-ribosyltransferase, SpyA (Korotkova et al., 2012). The microscopy analysis of 5005Δ*spyB* revealed long chains of unseparated cells. This observation indicates that the protein plays an important role in the regulation of cell division or separation in GAS. Furthermore, phenotypic screening uncovered decreased sensitivity of 5005Δ*spyB* to β-lactam antibiotics of the cephalosporin class and the cell wall hydrolase PlyC. In *Streptococcus* and *Staphylococcus* species, a defect in cell wall biosynthesis causes an altered susceptibility to β-lactam antibiotics (Berger-Bächi et al., 1989; Garcia-Bustos and Tomasz, 1990; Filipe et al., 2000; Filipe and Tomasz, 2000; Crisóstomo et al., 2006). Since cell wall synthesis and cell division are tightly linked cellular processes in bacteria, the function of SpyB could therefore be to modulate cell wall synthesis. To search for the SpyB-regulated step of cell wall synthesis, we examined the expression of the targets of β-lactam antibiotics, PBPs. These membrane proteins are involved in the last step of PG assembly. Using an assay with fluorescently labeled penicillin, we showed that 5005Δ*spyB* has no obvious defect in the expression of PBPs. Since β-lactam antibiotics compete with cell wall substrates for binding to PBPs, it is possible that the drug resistant phenotype of 5005Δ*spyB* arises from the perturbation of the cellular pool of cell wall precursors.

Using different hydrolytic conditions for analysis of cell wall composition, we found that the MGAS5005 cell wall is resistant to methanolysis. GAC is a major GAS cell wall component, and comprises about 40–60% of the total cell wall mass (Mistou et al., 2016). The GAC structure consists of a polyrhamnose backbone decorated with immunodominant GlcNA side-chains (Coligan et al., 1978). The simplest hypothesis for MGAS5005 cell wall resistance to methanolysis would be de-acetylation of GlcNA in GAC. Breaking a glycosidic bond between an amino sugar (i.e., de-acetylated GlcNA) and Rha would need stronger acidic conditions than hydrolysis of a bond between neutral sugars GlcNA and Rha (Merkle and Poppe, 1994; Jennings et al., 2015). This hypothesis of altered de-acetylation between the strains is in agreement with the strong hydrolysis data that demonstrate a significant increase in the recovery of monosaccharides from the MGAS5005 cell wall. Surprisingly, the cell wall of the 5005Δ*spyB* appeared to be susceptible to methanolysis as the recovery of monosaccharides was not significantly changed between the two hydrolytic conditions. These observations indicate structural differences in the cell wall between MGAS5005 and 5005Δ*spyB*, and suggest a role for SpyB in modification of GAC sugars. Moreover, our observation of increased binding of GlcNA-specific lectin to SpyB-deficient cells is an additional indication of the ability of SpyB to influence GlcNA amounts on the cell wall. Changes in N-acetylation were proposed in the study of *Pseudomonas aeruginosa* Pel exopolysaccharide, where it was revealed that Pel contains partially acetylated galactosamine and glucosamine sugars (Jennings et al., 2015). Similar to MGAS5005 GAC, the exopolysaccharide of the WT *P. aeruginosa* strain was resistant to mild acid hydrolysis and demonstrated an abundance of N-acetylgalactosamine and GlcNA after strong acid hydrolysis.

Rha-containing cell wall carbohydrate is produced by many species of the *Lactobaccillales* order (Mistou et al., 2016) and is covalently anchored to the peptidoglycan component MurNA (Deng et al., 2000; Swoboda et al., 2010). Most streptococcal species including GAS do not produce wall teichoic acid (WTA; Mistou et al., 2016), which is a major cell wall component of Gram positive bacteria, and instead synthesize a Rha-containing carbohydrate as a functional homolog of WTA (Caliot et al., 2012). In GAS and *Streptococcus agalactiae*, deletion of the genes essential for carbohydrate biosynthesis resulted in severe cell division abnormalities that include cell separation defects and aberrant septum placement (Caliot et al., 2012; van der Beek et al., 2015). Moreover, *S. agalactiae* group B specific carbohydrate deficient mutants demonstrated a reduced level of PG cross-linking and mislocalization of PG cell wall hydrolase PscB (CdhA homolog in *S. pyogenes*), which is required for PG cleavage during septation (Caliot et al., 2012). These phenotypes are reminiscent of the abnormalities reported in WTA-deficient strains of *Staphylococcus aureus* (Atilano et al., 2010; Schlag et al., 2010; Campbell et al., 2011) and *Bacillus subtilis* (Bhavsar et al., 2001). It is possible that the increased aggregation of SpyB-deficient cells observed in our study is also due to mislocalization of PG cell wall hydrolase CdhA (Pancholi et al., 2010). Interestingly, this SpyB phenotype corresponds to a previous description of an *S. pyogenes* mutant of the glycosyltransferase *gacI*, which is defective for GlcNA side-chain addition to the Rha backbone of GAC. The *gacI* mutant displayed a defect in the chain separation process, suggesting an important role for GAC composition in cell morphological processes (van Sorge et al., 2014).

Assigning SpyB a role in the modulation of GAC composition raises the question whether SpyB function is regulated in *S*. *pyogenes*. Our *in vitro* studies demonstrated that SpyB interacts with porphyrins, and hemin, the iron-chelated form of porphyrin, is preferable for binding. GAS lacks a heme biosynthetic pathway and host-derived heme is the preferred source of nutrient iron during infection (Lei et al., 2002; Bates et al., 2003). Heme released from erythrocytes and tissue by GAS hemolytic toxins is transported inside bacteria by systems dedicated to the acquisition of heme from host hemoproteins (Lei et al., 2002, 2003; Bates et al., 2003; Ouattara et al., 2010). While heme is an important iron source for GAS during infection, high concentrations of heme causes toxicity (Sachla et al., 2014). It has been reported that exposing GAS to sub-lethal concentrations of heme results in oxidative damage to membrane lipids, DNA and proteins (Sachla et al., 2014; Sachla and Eichenbaum, 2016). The fact that SpyB binds hemin with a dissociation constant in the low micromolar range suggests a role for SpyB in heme sensing, storage or trafficking rather than in heme scavenging from host hemoproteins. We showed that hemin binding induces SpyB dimerization, which involves a disulfide bond between monomers. Since heme is present at physiological pH predominantly as a dimer (Asher et al., 2009), oligomerization of SpyB may occur around the heme dimer, where two molecules of heme are cofacialy stacked. Interestingly, hemin-induced oligomerization has been reported for other proteins including hemophore-like protein HusA from *Porphyromonas gingivalis* (Gao et al., 2010), CnaN lipoprotein from *Campylobacter jejuni* (Chan et al., 2006), neuronal nitric oxide synthase (Klatt et al., 1996), RNA-binding protein DGCR8 (Faller et al., 2007) and human cytoplasmic arginyl-tRNA synthetase (Yang et al., 2010). Moreover, heme-induced oligomerization is important for the function of some of these heme-binding proteins (Klatt et al., 1996; Faller et al., 2007; Yang et al., 2010). It remains to be determined whether hemin binds SpyB *in vivo* and how it affects SpyB function. Our quantitative RT-PCR data demonstrated that hemin did not affect the transcription of *spyAB* operon (Figure S2B). Moreover, hemin exposure did not modify the growth phenotypes of MGAS5005, 5005Δ*spyB*, and 5005Δ*spyA* in nutrient rich medium (Figure S10).

Another exciting possibility is the regulation of SpyB function by SpyA. SpyA, an enigmatic toxin-like protein has been implicated in GAS virulence (Hoff et al., 2011; Lin et al., 2015). However, SpyA has unusual localization for a toxin. It is attached to the GAS membrane via a short N-terminal hydrophobic domain with the functional domain in the extracellular space, suggesting its role is in post-translational regulation of GAS extracellular proteins (Korotkova et al., 2012). Similar to heme, NAD could be released from host cells by GAS as a result of secretion of hemolytic toxins. SpyA might use the extracellular NAD for ADP-ribosylation of SpyB leading to modulation of its function. Thus, SpyB function might be regulated by two host-derived molecules: heme and NAD. Interestingly, the phenotypic characterization of 5005Δ*spyA* did not reveal the phenotypes observed for 5005Δ*spyB*. This could be explained by a lack of extracellular NAD that would be required for SpyA ADP-ribosyltransferase activity. Whether NAD and SpyA influence SpyB function is an area of our active investigation.

In conclusion, our study provides a framework for a thorough investigation of SpyB function in the regulation of GAC composition. Identification of a physiological role for SpyB in *S. pyogenes* will illuminate SpyA function and help to solve the puzzle of how bacteria sense and respond to multiple environmental stimuli that occur during the course of infection.

## Author contributions

Conceived and designed the experiments: RE, JC, JR, LF, PA, ES, HZ, KK, VP, NK. Performed the experiments: RE, JC, SK, ER, JR, LF, BJ, VT, VP, NK. Analyzed the data: RE, JC, ER, JR, LF, BJ, PA, VT, KK, VP, NK. Wrote the paper: RE, NK.

### Conflict of interest statement

The authors declare that the research was conducted in the absence of any commercial or financial relationships that could be construed as a potential conflict of interest.

## References

[B1] Agarwal S. Agarwal S. Pancholi P. Pancholi V. (2011). Role of serine/threonine phosphatase (SP-STP) in *Streptococcus pyogenes* physiology and virulence. J. Biol. Chem. 286, 41368–41380. 10.1074/jbc.M111.286690 21917918 PMC3308849

[B2] Albarracin Orio A. G. Pinas G. E. Cortes P. R. Cian M. B. Echenique J. (2011). Compensatory evolution of pbp mutations restores the fitness cost imposed by beta-lactam resistance in *Streptococcus pneumoniae*. PLoS Pathog. 7:e1002000. 10.1371/journal.ppat.1002000 21379570 PMC3040684

[B3] Ascoli F. Fanelli M. R. Antonini E. (1980). Preparation and properties of apohemoglobin and reconstituted hemoglobins. Methods Enzymol. 76, 72–87. 7329287 10.1016/0076-6879(81)76115-9

[B4] Asher C. de Villiers K. A. Egan T. J. (2009). Speciation of ferriprotoporphyrin IX in aqueous and mixed aqueous solution is controlled by solvent identity, pH, and salt concentration. Inorg. Chem. 48, 7994–8003. 10.1021/ic900647y 19572726

[B5] Atilano M. L. Pereira P. M. Yates J. Reed P. Veiga H. Pinho M. G.. (2010). Teichoic acids are temporal and spatial regulators of peptidoglycan cross-linking in *Staphylococcus aureus*. Proc. Natl. Acad. Sci. U.S.A. 107, 18991–18996. 10.1073/pnas.1004304107 20944066 PMC2973906

[B6] Bates C. S. Montañez G. E. Woods C. R. Vincent R. M. Eichenbaum Z. (2003). Identification and characterization of a *Streptococcus pyogenes* operon involved in binding of hemoproteins and acquisition of iron. Infect. Immun. 71, 1042–1055. 10.1128/IAI.71.3.1042-1055.2003 12595414 PMC148835

[B7] Berger-Bächi B. Barberis-Maino L. Strässle A. Kayser F. H. (1989). FemA, a host-mediated factor essential for methicillin resistance in *Staphylococcus aureus*: molecular cloning and characterization. Mol. Gen. Genet. 219, 263–269. 2559314 10.1007/BF00261186

[B8] Berry E. A. Trumpower B. L. (1987). Simultaneous determination of hemes a, b, and c from pyridine hemochrome spectra. Anal. Biochem. 161, 1–15. 3578775 10.1016/0003-2697(87)90643-9

[B9] Bhavsar A. P. Beveridge T. J. Brown E. D. (2001). Precise deletion of tagD and controlled depletion of its product, glycerol 3-phosphate cytidylyltransferase, leads to irregular morphology and lysis of *Bacillus subtilis* grown at physiological temperature. J. Bacteriol. 183, 6688–6693. 10.1128/JB.183.22.6688-6693.2001 11673441 PMC95502

[B10] Bowman S. E. Bren K. L. (2008). The chemistry and biochemistry of heme c: functional bases for covalent attachment. Nat. Prod. Rep. 25, 1118–1130. 10.1039/b717196j 19030605 PMC2654777

[B11] Bui N. K. Eberhardt A. Vollmer D. Kern T. Bougault C. Tomasz A.. (2012). Isolation and analysis of cell wall components from *Streptococcus pneumoniae*. Anal. Biochem. 421, 657–666. 10.1016/j.ab.2011.11.026 22192687

[B12] Caliot É. Dramsi S. Chapot-Chartier M. P. Courtin P. Kulakauskas S. Péchoux C.. (2012). Role of the Group B antigen of *Streptococcus agalactiae*: a peptidoglycan-anchored polysaccharide involved in cell wall biogenesis. PLoS Pathog. 8:e1002756. 10.1371/journal.ppat.1002756 22719253 PMC3375309

[B13] Campbell J. Singh A. K. Santa Maria J. P. Jr. Kim Y. Brown S. Swoboda J. G.. (2011). Synthetic lethal compound combinations reveal a fundamental connection between wall teichoic acid and peptidoglycan biosyntheses in *Staphylococcus aureus*. ACS Chem. Biol. 6, 106–116. 10.1021/cb100269f 20961110 PMC3025082

[B14] Caparon M. G. Scott J. R. (1991). Genetic manipulation of pathogenic streptococci. Methods Enzymol. 204, 556–586. 1658571 10.1016/0076-6879(91)04028-m

[B15] Carapetis J. R. Steer A. C. Mulholland E. K. Weber M. (2005). The global burden of group A streptococcal diseases. Lancet Infect. Dis. 5, 685–694. 10.1016/S1473-3099(05)70267-X 16253886

[B16] Chambers R. E. Clamp J. R. (1971). An assessment of methanolysis and other factors used in the analysis of carbohydrate-containing materials. Biochem. J. 125, 1009–1018. 5144210 10.1042/bj1251009 PMC1178263

[B17] Chan A. C. Lelj-Garolla B. Rosell F. I. Pedersen K. A. Mauk A. G. Murphy M. E. P. (2006). Cofacial heme binding is linked to dimerization by a bacterial heme transport protein. J. Mol. Biol. 362, 1108–1119. 10.1016/j.jmb.2006.08.001 16950397

[B18] Chang J. C. LaSarre B. Jimenez J. C. Aggarwal C. Federle M. J. (2011). Two group A streptococcal peptide pheromones act through opposing Rgg regulators to control biofilm development. PLoS Pathog. 7:e1002190. 10.1371/journal.ppat.1002190 21829369 PMC3150281

[B19] Coligan J. E. Kindt T. J. Krause R. M. (1978). Structure of the streptococcal groups A, A-variant and C carbohydrates. Immunochemistry 15, 755–760. 85600 10.1016/0161-5890(78)90105-0

[B20] Coye L. H. Collins C. M. (2004). Identification of SpyA, a novel ADP-ribosyltransferase of *Streptococcus pyogenes*. Mol. Microbiol. 54, 89–98. 10.1111/j.1365-2958.2004.04262.x 15458407

[B21] Crisóstomo M. I. Vollmer W. Kharat A. S. Inhülsen S. Gehre F. Buckenmaier S.. (2006). Attenuation of penicillin resistance in a peptidoglycan O-acetyl transferase mutant of *Streptococcus pneumoniae*. Mol. Microbiol. 61, 1497–1509. 10.1111/j.1365-2958.2006.05340.x 16968223

[B22] Deng L. Kasper D. L. Krick T. P. Wessels M. R. (2000). Characterization of the linkage between the type III capsular polysaccharide and the bacterial cell wall of group B Streptococcus. J. Biol. Chem. 275, 7497–7504. 10.1074/jbc.275.11.7497 10713053

[B23] DuBois M. Gilles K. A. Hamilton J. K. Rebers P. A. Smith F. (1956). Colorimetric method for determination of sugars and related substances. Anal. Chem. 28, 350. 10.1038/168167a0 14875032

[B24] Dunstan R. A. Heinz E. Wijeyewickrema L. C. Pike R. N. Purcell A. W. Evans T. J.. (2013). Assembly of the type II secretion system such as found in *Vibrio cholerae* depends on the novel Pilotin AspS. PLoS Pathog. 9:e1003117. 10.1371/journal.ppat.1003117 23326233 PMC3542185

[B25] Falaleeva M. Zurek O. W. Watkins R. L. Reed R. W. Ali H. Sumby P.. (2014). Transcription of the *Streptococcus pyogenes* hyaluronic acid capsule biosynthesis operon is regulated by previously unknown upstream elements. Infect. Immun. 82, 5293–5307. 10.1128/IAI.02035-14 25287924 PMC4249290

[B26] Faller M. Matsunaga M. Yin S. Loo J. A. Guo F. (2007). Heme is involved in microRNA processing. Nat. Struct. Mol. Biol. 14, 23–29. 10.1038/nsmb1182 17159994

[B27] Filipe S. R. Severina E. Tomasz A. (2000). Distribution of the mosaic structured murM genes among natural populations of *Streptococcus pneumoniae*. J. Bacteriol. 182, 6798–6805. 10.1128/JB.182.23.6798-6805.2000 11073926 PMC111424

[B28] Filipe S. R. Tomasz A. (2000). Inhibition of the expression of penicillin resistance in *Streptococcus pneumoniae* by inactivation of cell wall muropeptide branching genes. Proc. Natl. Acad. Sci. U.S.A. 97, 4891–4896. 10.1073/pnas.080067697 10759563 PMC18328

[B29] Gao J. L. Nguyen K. A. Hunter N. (2010). Characterization of a hemophore-like protein from *Porphyromonas gingivalis*. J. Biol. Chem. 285, 40028–40038. 10.1074/jbc.M110.163535 20940309 PMC3000985

[B30] Garcia-Bustos J. Tomasz A. (1990). A biological price of antibiotic resistance: major changes in the peptidoglycan structure of penicillin-resistant pneumococci. Proc. Natl. Acad. Sci. U.S.A. 87, 5415–5419. 2371278 10.1073/pnas.87.14.5415 PMC54335

[B31] Hakenbeck R. Bruckner R. Denapaite D. Maurer P. (2012). Molecular mechanisms of beta-lactam resistance in *Streptococcus pneumoniae*. Future Microbiol. 7, 395–410. 10.2217/fmb.12.2 22393892

[B32] Hoff J. S. Dewald M. Moseley S. L. Collins C. M. Voyich J. M. (2011). SpyA, a C3-like ADP-ribosyltransferase, contributes to virulence in a mouse subcutaneous model of *Streptococcus pyogenes* infection. Infect. Immun. 79, 2404–2411. 10.1128/IAI.01191-10 21422178 PMC3125853

[B33] Huang D. H. Rama Krishna N. Pritchard D. G. (1986). Characterization of the group A streptococcal polysaccharide by two-dimensional 1H-nuclear-magnetic-resonance spectroscopy. Carbohydr. Res. 155, 193–199. 3539332 10.1016/s0008-6215(00)90145-9

[B34] Icenogle L. M. Hengel S. M. Coye L. H. Streifel A. Collins C. M. Goodlett D. R.. (2012). Molecular and biological characterization of Streptococcal SpyA-mediated ADP-ribosylation of intermediate filament protein vimentin. J. Biol. Chem. 287, 21481–21491. 10.1074/jbc.M112.370791 22549780 PMC3375569

[B35] Jennings L. K. Storek K. M. Ledvina H. E. Coulon C. Marmont L. S. Sadovskaya I.. (2015). Pel is a cationic exopolysaccharide that cross-links extracellular DNA in the *Pseudomonas aeruginosa* biofilm matrix. Proc. Natl. Acad. Sci. U.S.A. 112, 11353–11358. 10.1073/pnas.1503058112 26311845 PMC4568648

[B36] Kant S. Agarwal S. Pancholi P. Pancholi V. (2015). The *Streptococcus pyogenes* orphan protein tyrosine phosphatase, SP-PTP, possesses dual specificity and essential virulence regulatory functions. Mol. Microbiol. 97, 515–540. 10.1111/mmi.13047 25939957

[B37] Klatt P. Pfeiffer S. List B. M. Lehner D. Glatter O. Bächinger H. P.. (1996). Characterization of heme-deficient neuronal nitric-oxide synthase reveals a role for heme in subunit dimerization and binding of the amino acid substrate and tetrahydrobiopterin. J. Biol. Chem. 271, 7336–7342. 8631754 10.1074/jbc.271.13.7336

[B38] Kocaoglu O. Calvo R. A. Sham L. T. Cozy L. M. Lanning B. R. Francis S.. (2012). Selective penicillin-binding protein imaging probes reveal substructure in bacterial cell division. ACS Chem. Biol. 7, 1746–1753. 10.1021/cb300329r 22909777 PMC3663142

[B39] Kocaoglu O. Tsui H. C. Winkler M. E. Carlson E. E. (2015). Profiling of beta-lactam selectivity for penicillin-binding proteins in *Streptococcus pneumoniae* D39. Antimicrob. Agents Chemother. 59, 3548–3555. 10.1128/AAC.05142-14 25845878 PMC4432181

[B40] Korotkova N. Hoff J. S. Becker D. M. Quinn J. K. Icenogle L. M. Moseley S. L. (2012). SpyA is a membrane-bound ADP-ribosyltransferase of *Streptococcus pyogenes* which modifies a streptococcal peptide, SpyB. Mol. Microbiol. 83, 936–952. 10.1111/j.1365-2958.2012.07979.x 22288436 PMC3288127

[B41] Lei B. Liu M. Voyich J. M. Prater C. I. Kala S. V. DeLeo F. R.. (2003). Identification and characterization of HtsA, a second heme-binding protein made by *Streptococcus pyogenes*. Infect. Immun. 71, 5962–5969. 10.1128/IAI.71.10.5962-5969.2003 14500516 PMC201091

[B42] Lei B. Smoot L. M. Menning H. M. Voyich J. M. Kala S. V. Deleo F. R.. (2002). Identification and characterization of a novel heme-associated cell surface protein made by *Streptococcus pyogenes*. Infect. Immun. 70, 4494–4500. 10.1128/IAI.70.8.4494-4500.2002 12117961 PMC128137

[B43] Lin A. E. Beasley F. C. Keller N. Hollands A. Urbano R. Troemel E. R.. (2015). A group A Streptococcus ADP-ribosyltransferase toxin stimulates a protective interleukin 1beta-dependent macrophage immune response. MBio 6:e00133. 10.1128/mBio.00133-15 25759502 PMC4453525

[B44] Merkle R. K. Poppe I. (1994). Carbohydrate composition analysis of glycoconjugates by gas-liquid chromatography/mass spectrometry. Meth. Enzymol. 230, 1–15. 8139491 10.1016/0076-6879(94)30003-8

[B45] Mistou M.-Y. Sutcliffe I. C. Van Sorge N. M. (2016). Bacterial glycobiology: rhamnose-containing cell wall polysaccharides in Gram-positive bacteria. FEMS Microbiol. Rev. 40, 464–479. 10.1093/femsre/fuw006 26975195 PMC4931226

[B46] Nelson D. Loomis L. Fischetti V. A. (2001). Prevention and elimination of upper respiratory colonization of mice by group A streptococci by using a bacteriophage lytic enzyme. Proc. Natl. Acad. Sci. U.S.A. 98, 4107–4112. 10.1073/pnas.061038398 11259652 PMC31187

[B47] Nelson D. Schuch R. Chahales P. Zhu S. Fischetti V. A. (2006). PlyC: a multimeric bacteriophage lysin. Proc. Natl. Acad. Sci. U.S.A. 103, 10765–10770. 10.1073/pnas.0604521103 16818874 PMC1487170

[B48] Ortiz de Orué Lucana D. Fedosov S. N. Wedderhoff I. Che E. N. Torda A. E. (2014). The extracellular heme-binding protein HbpS from the soil bacterium *Streptomyces reticuli* is an aquo-cobalamin binder. J. Biol. Chem. 289, 34214–34228. 10.1074/jbc.M114.585489 25342754 PMC4256353

[B49] Ouattara M. Cunha E. B. Li X. Huang Y. S. Dixon D. Eichenbaum Z. (2010). Shr of group A Streptococcus is a new type of composite NEAT protein involved in sequestering haem from methaemoglobin. Mol. Microbiol. 78, 739–756. 10.1111/j.1365-2958.2010.07367.x 20807204 PMC2963705

[B50] Pancholi V. Boël G. Jin H. (2010). *Streptococcus pyogenes* Ser/Thr kinase-regulated cell wall hydrolase is a cell division plane-recognizing and chain-forming virulence factor. J. Biol. Chem. 285, 30861–30874. 10.1074/jbc.M110.153825 20643653 PMC2945579

[B51] Philippe J. Vernet T. Zapun A. (2014). The elongation of ovococci. Microb. Drug Resist. 20, 215–221. 10.1089/mdr.2014.0032 24773288 PMC4050454

[B52] Price L. B. Johnson J. R. Aziz M. Clabots C. Johnston B. Tchesnokova V.. (2013). The epidemic of extended-spectrum-beta-lactamase-producing *Escherichia coli* ST131 is driven by a single highly pathogenic subclone, H30-Rx. MBio 4:e00377-13. 10.1128/mBio.00377-13 24345742 PMC3870262

[B53] Raz A. Fischetti V. A. (2008). Sortase A localizes to distinct foci on the *Streptococcus pyogenes* membrane. Proc. Natl. Acad. Sci. U.S.A. 105, 18549–18554. 10.1073/pnas.0808301105 19017791 PMC2587614

[B54] Sachla A. J. Eichenbaum Z. (2016). The GAS PefCD exporter is a MDR system that confers resistance to heme and structurally diverse compounds. BMC Microbiol. 16:68. 10.1186/s12866-016-0687-6 27095127 PMC4837585

[B55] Sachla A. J. Le Breton Y. Akhter F. Mciver K. S. Eichenbaum Z. (2014). The crimson conundrum: heme toxicity and tolerance in GAS. Front. Cell. Infect. Microbiol. 4:159. 10.3389/fcimb.2014.00159 25414836 PMC4220732

[B56] Santander J. Martin T. Loh A. Pohlenz C. Gatlin D. M. III Curtiss R. III. (2013). Mechanisms of intrinsic resistance to antimicrobial peptides of *Edwardsiella ictaluri* and its influence on fish gut inflammation and virulence. Microbiology 159, 1471–1486. 10.1099/mic.0.066639-0 23676433 PMC4085987

[B57] Schlag M. Biswas R. Krismer B. Kohler T. Zoll S. Yu W.. (2010). Role of staphylococcal wall teichoic acid in targeting the major autolysin Atl. Mol. Microbiol. 75, 864–873. 10.1111/j.1365-2958.2009.07007.x 20105277

[B58] Shepherd M. Heath M. D. Poole R. K. (2007). NikA binds heme: a new role for an *Escherichia coli* periplasmic nickel-binding protein. Biochemistry 46, 5030–5037. 10.1021/bi700183u 17411076

[B59] Storz G. Wolf Y. I. Ramamurthi K. S. (2014). Small proteins can no longer be ignored. Annu. Rev. Biochem. 83, 753–777. 10.1146/annurev-biochem-070611-102400 24606146 PMC4166647

[B60] Sumby P. Porcella S. F. Madrigal A. G. Barbian K. D. Virtaneva K. Ricklefs S. M.. (2005). Evolutionary origin and emergence of a highly successful clone of serotype M1 group a Streptococcus involved multiple horizontal gene transfer events. J. Infect. Dis. 192, 771–782. 10.1086/432514 16088826

[B61] Swoboda J. G. Campbell J. Meredith T. C. Walker S. (2010). Wall teichoic acid function, biosynthesis, and inhibition. Chembiochem 11, 35–45. 10.1002/cbic.200900557 19899094 PMC2798926

[B62] Thomas P. E. Ryan D. Levin W. (1976). An improved staining procedure for the detection of the peroxidase activity of cytochrome P-450 on sodium dodecyl sulfate polyacrylamide gels. Anal. Biochem. 75, 168–176. 822747 10.1016/0003-2697(76)90067-1

[B63] Treviño J. Liu Z. Cao T. N. Ramirez-Peña E. Sumby P. (2013). RivR is a negative regulator of virulence factor expression in group A Streptococcus. Infect. Immun. 81, 364–372. 10.1128/IAI.00703-12 23147037 PMC3536152

[B64] van der Beek S. L. Le Breton Y. Ferenbach A. T. Chapman R. N. Van Aalten D. M. Navratilova I.. (2015). GacA is essential for Group A Streptococcus and defines a new class of monomeric dTDP-4-dehydrorhamnose reductases (RmlD). Mol. Microbiol. 98, 946–962. 10.1111/mmi.13169 26278404 PMC4832382

[B65] van Sorge N. M. Cole J. N. Kuipers K. Henningham A. Aziz R. K. Kasirer-Friede A.. (2014). The classical lancefield antigen of group a Streptococcus is a virulence determinant with implications for vaccine design. Cell Host Microbe 15, 729–740. 10.1016/j.chom.2014.05.009 24922575 PMC4078075

[B66] Yang F. Xia X. Lei H. Y. Wang E. D. (2010). Hemin binds to human cytoplasmic arginyl-tRNA synthetase and inhibits its catalytic activity. J. Biol. Chem. 285, 39437–39446. 10.1074/jbc.M110.159913 20923763 PMC2998122

[B67] Yin L. Dragnea V. Bauer C. E. (2012). PpsR, a regulator of heme and bacteriochlorophyll biosynthesis, is a heme-sensing protein. J. Biol. Chem. 287, 13850–13858. 10.1074/jbc.M112.346494 22378778 PMC3340169

[B68] Yin L. Dragnea V. Feldman G. Hammad L. A. Karty J. A. Dann C. E. III. (2013). Redox and light control the heme-sensing activity of AppA. MBio 4:e00563-13. 10.1128/mBio.00563-13 23982072 PMC3760249

