# Supplementary material for: SpyB, a Small Heme-Binding Protein, Affects the Composition of the Cell Wall in Streptococcus pyogenes
Source: Front Cell Infect Microbiol. 2016 Oct 13;6:126. doi: 10.3389/fcimb.2016.00126 (PMC5061733; doi:10.3389/fcimb.2016.00126)
Supplement: Supplementary file 1 [file Image1.pdf]

Table S1. Bacterial strains and plasmids

Strain or plasmid	Description	Reference
S. pyogenes		
MGAS5005	Wild-type, M1-serotype strain	(Sumby et al., 2005)
5005Δ*spyA*	*spyA* deletion mutant	(Hoff et al., 2011)
5005Δ*spyB*	*spyB* deletion mutant	This study
5005Δ*spyB spyB*$^{+}$	*spyB* deletion mutant complemented with *spyB* in *cis*	This study
E. coli		
DH5α	Cloning host	Invitrogen
Rosetta (DE3)	Protein expression host	Novagen
Plasmids		
pBBL740	*S. pyogenes* integrational plasmid	(Zhu et al., 2009)
pBBL740*spyB*	pBBL740 containing a 1.4 kbp DNA fragment carrying the *spyB* locus and regions flanking either side	This study
pBBL740Δs*pyB*	pBBL740*spyB* derived plasmid containing a *spyB* in frame deletion	This study
p*spyBs*	pBBL740*spyB* derived plasmid containing a synonymous mutation in Arg 9 of *spyB*.	This study
pET-22b(+)	*E. coli* protein expression vector harboring T7 promoter	Novagen
pmalE	pET-22b(+) derived plasmid containing *malE* followed by a multiple cloning site and the sequence encoding a His-tag	This study
pmalEspyB	pmalE derived plasmid containing *spyB* fused at the N-terminus with *malE* and at the C-terminus with a TEV protease recognition site followed by a His-tag sequence.	This study
pKV1111	pET-22b(+) derived plasmid containing *malE*	(Dunstan et al., 2013)

Table S2. General primers

Primer	Sequence[a]
spyB-BamHI-f	CGATCGGATCCCTGTTTCTAATGGATCACGTTTATG
spyB-XhoI-r	GCGAGCTCGAGGCTGACAGCCTCTTGGAAACAAG
spyArt-f	GTACACGCTACACCAAGCAC
spyArt-r	GACACAGATGCGCACCTCC
plr-f	CGTCTTGCATTCCGCCGTATTC
plr-r	GATCTGTAAGGTCATTGATACG
5005-check-f	GGGATGAGCTATATACCAAC
5005-check-r	GACATTCGTTCTAGTTTAGG
spyB-NcoI-f	CACGTCCATGGGCACAAAAAGATTAGCCTGTCTG
spyB-TEV-XhoI-r	CTCGATCTCGAGGCCCTGAAAATACAGGTTCTCGCATAGTTCCCCCTGACACCAG

[a] - Restriction sites are underlined.

Table S3. Primers for site-directed mutagenesis

primer	sequence	Introduced mutations
spyBΔ-f<br>spyBΔ-r	CAAAAAGATTAGCCTGTCTGCTTTTGGGCTGGTGTCAG<br>CTGACACCAGCCCAAAAGCAGACAGGCTAATCTTTTTG	In frame truncation of *spyB* 51 bp (deletion of 9-25 amino acids)
SpyBwt-f<br>SpyBwt-r	GACAAAAAGATTAGCCTGTCTGCGAAACTGGTGGTGTCAAGAGCTAGCC<br>GGCTAGCTCTTGACACCACCAGTTTCGCAGACAGGCTAATCTTTTTGTC	A synonymous mutation in R9 of *spyB*
C30A-f<br>C30A-r	GGGTCAGCCTTTTGGGCTGGGCTCAGGGGGAACTATGCGAG<br>CTCGCATAGTTCCCCCTGAGCCCAGCCCAAAAGGCTGACCC	C30A mutation in *spyB*
C30-35A-f<br>C30-35A-r	GCTGGGCTCAGGGGGAACTAGCCGAGAACCTGTATTTTCAGGGC<br>GCCCTGAAAATACAGGTTCTCGGCTAGTTCCCCCTGAGCCCAGC	C30A and C35A mutations in *spyB*
C13A-f<br>C13A-r	CGCTCTGAGAAACTGGTGGGCTCAAGAGCTAGCCAAAAGGTC<br>GACCTTTTGGCTAGCTCTTGAGCCCACCAGTTTCTCAGAGCG	C13A mutation in *spyB*
C7A-f<br>C7A-r	GACAAAAAGATTAGCCGCTCTGAGAAACTGGTGG<br>CCACCAGTTTCTCAGAGCGGCTAATCTTTTTGTC	C7A mutation in *spyB*

Table S4. PBPs predicted in *S. pyogenes*

PBP	Gene[b]	aa	MW, kDa	Homolog[d]	Identity, %	Putative domain structure[e]
PBP1A	Spy1355	721	80.2	PBP1A(Spn)	61	TM-TG-TP
PBP1B_α[a]	Spy0082	770	84.5	PBP1B(Spn)	59	TM-TG-TP
PBP1B_β[a]		766	83.9			
PBP1B_γ[a]		765	83.8			
PBP2A_α[a]	Spy1753	778	85.7	PBP2A(Spn)	59	TM-TG-TP
PBP2A_β[a]		694	75.8			
PBP2X	Spy1366	751	82.8	PBP2X(Spn)	54	TM-D-TP-PASTA
PBP3A	Spy0248	410	43.4[c]	PBP3(Spn)	47	SP-CP-A
PBP3B	Spy0247	393	41.1[c]	PBP4(Sau)	38	SP-CP-TM
PBP3C	Spy0817	415	42.6[c]	PBP3(Spn)	35	SP-CP-A

[a] Examination of PBP-encoding sequences in MGAS5005 genome identified PBP1B and PBP2A transcripts as leaderless (Moll et al., 2002) that carry start codons at their 5′ terminus. Furthermore, both transcripts displayed possible alternate start codons that are preceded by plausible Shine-Dalgarno sequences Sequence analysis of PBP1B and PBP2A-encoding genes in the genomes of other streptococcus species revealed that leaderless and alternative start codons are common features of these genes in streptococci. This observation suggests that PBP1B and PBP2A might be present in the cell membrane in several molecular forms that we termed PBP1B_α, PBP1B_β, PBP1B_γ, PBP2A_α and PBP2A_β;

[b] Spy numbers from MGAS5005 genome;

[c] MW corresponds to mature protein;

[d] Spn is *Streptococcus pneumoniae* and Sau is *Staphylococcus aureus;*

[e] TM, transmembrane domain; TG, transglycosylase domain; TP, transpeptidase domain; D, interaction domain; PASTA, PASTA domain; SP, signal peptide; CP, carboxypeptidase domain; A, amphipathic helix domain.

Table S5. Concentration of total carbohydrates in bacteria

**Strains**	**nM CHO/µg of protein**
MGAS5005	0.1059± 0.0024
5005*ΔspyB*	0.1094± 0.0045

Table S6. Glycosyl composition analysis of MGAS5005 and 5005Δ*spyB* cell wall

		mild hydrolysis			harsh hydrolysis		
	residue	weight [μg CHO[1]/ 100μg]	nmol [nmol CHO/ 100μg]	mol [%]	weight [μg CHO/ 100μg]	nmol [nmol CHO/ 100μg]	mol [%]
MGAS5005	Rha	24.0	1462.4	89.7	50.9	3101.4	83.9
	GlcNA	3.7	168.7	10.3	13.0	586.6	15.9
	MurNA	0.0	0.0	0.0	0.2	10.3	0.3
5005Δ*spyB*	Rha	37.6	2289.9	81.8	22.1	1347.8	75.2
	GlcNA	11.3	509.5	18.2	9.7	437.0	24.4
	MurNA	0.0	0.0	0.0	0.2	8.5	0.5

[1]CHO – carbohydrate

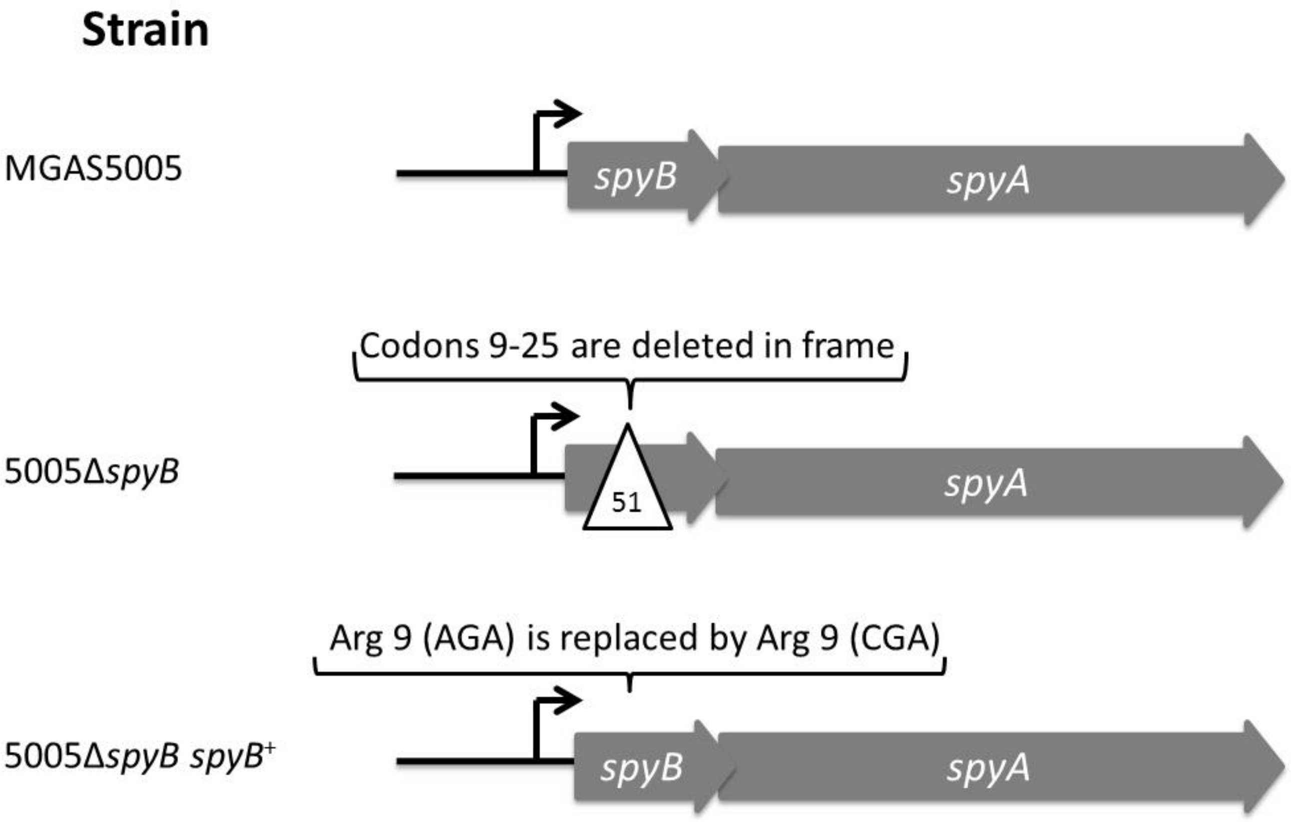

Figure S1. Schematic representations of the construction of the mutants used in this study. Bent arrows indicate the promoter of *spyBA* operon. The open triangle depicts the deletion that was constructed. The number of deleted nucleotides is indicated inside the triangle. A bracket denotes changes in the amino acid sequence of SpyB.

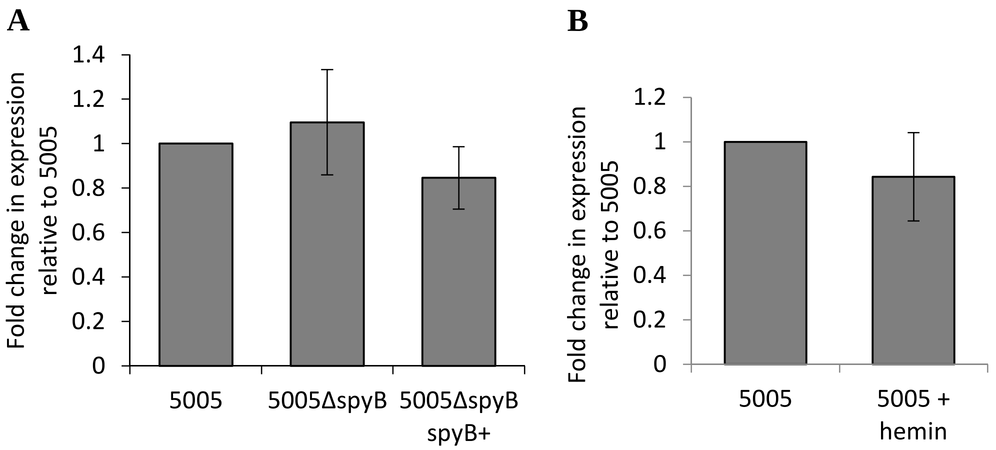

Figure S2. RT-qPCR analysis of expression from *spyA*. RT-qPCR analysis was performed on cDNA from (**A**) MGAS5005, 5005Δ*spyB* and 5005Δ*spyB spyB*$^{+}$ and (**B**) MGAS5005 grown in the absence and presence of 2 μM hemin. The mean fold change in *spyA* transcript, relative to the housekeeping gene *plr*, is presented. The fold changes are relative to MGAS5005, which was converted to 1. The data are from triplicate experiments ± standard deviation. There was no significant difference in expression as determined by the Student's *t*-test.

A

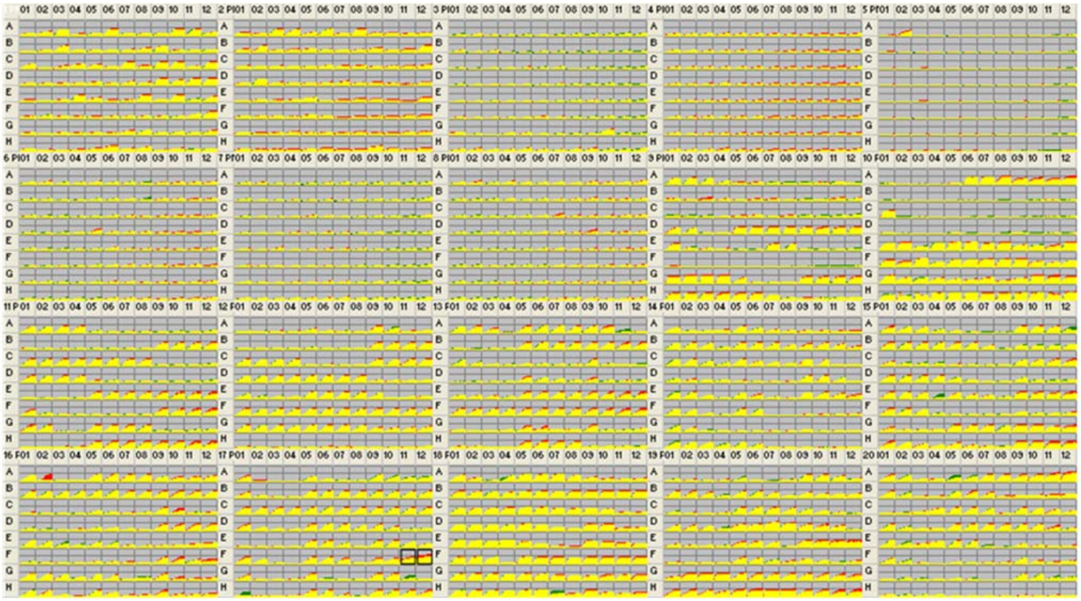

B

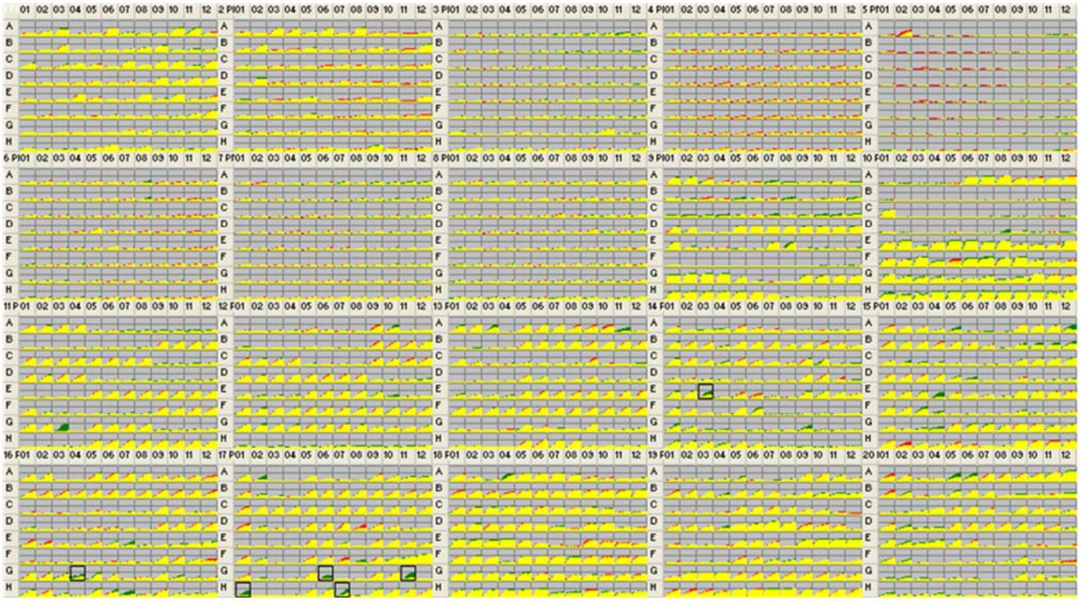

C

	Phenotypes gained	Phenotypes lost	Target/function
5005Δ*spyA*		Tannic acid	Antimicrobial from plants
5005Δ*spyB*	Caffeine		cAMP phosphodiesterase
	Chromium (III) chloride		Toxic cation
	Cefoperazone		Cell wall cephalosporin
	Cefamandole nafate		Cell wall cephalosporin
	Cefsulodin		Cell wall cephalosporin
	Cefoxitin		Cell wall cephalosporin

Figure S3. Phenotype MicroArray data for MGAS5005 vs 5005Δ*spyB* and 5005Δ*spyA*. Growth phenotypes of MGAS5005 vs 5005Δ*spyA* (**A**) and MGAS5005 vs 5005Δ*spyB* (**B**) were assessed using the Biolog plates at Biolog's PM Services facility. A total of 20 96-well PM plates constituting eight metabolic panels (PM1 to PM8); and 12 sensitivity panels (PM9 to PM20) were used. Two replicates were conducted for each strain and the consensus plots are presented. (**C**) Phenotypes gained and lost for 5005Δ*spyA* and 5005Δ*spyB*

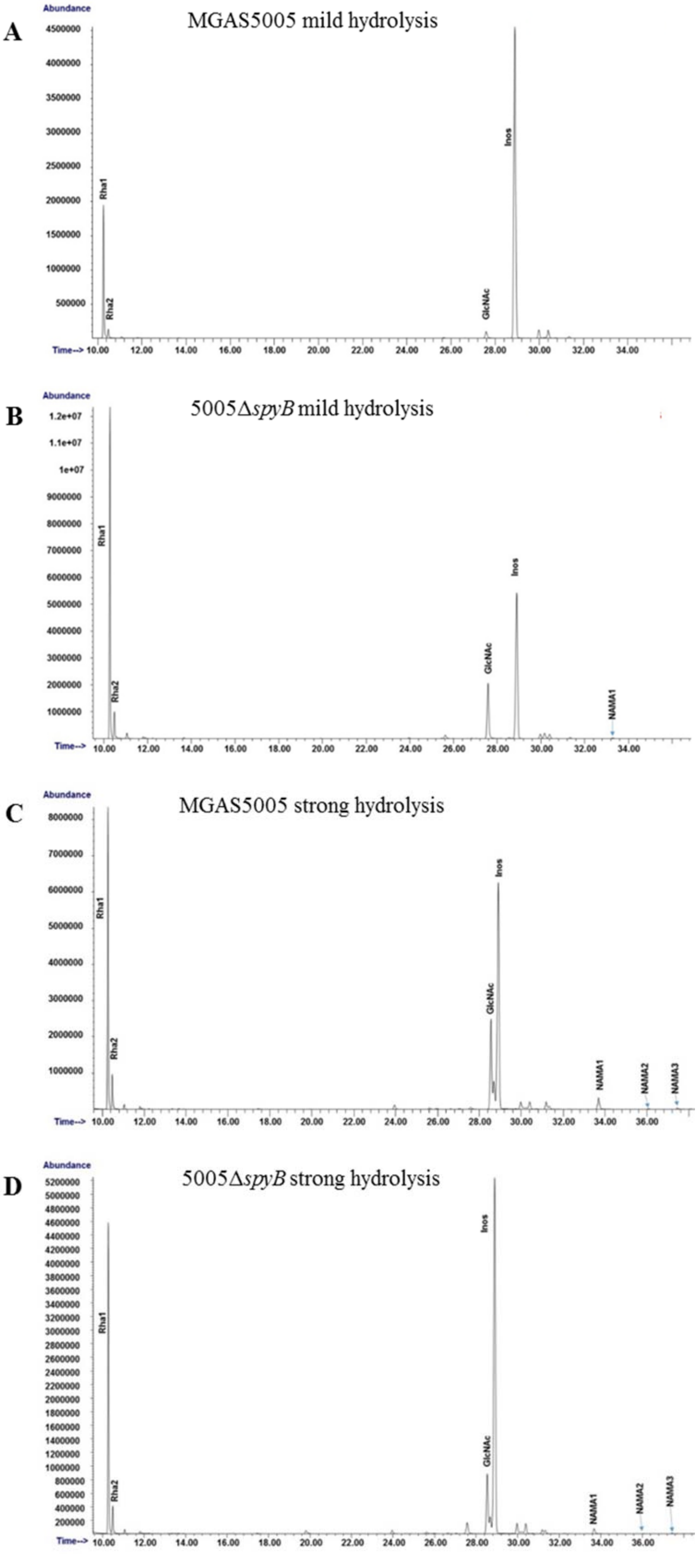
A
MGAS5005 mild hydrolysis
Abundance
Time-->
Rha1
Rha2
GlcNAc
Inos
B
5005Δ*spyB* mild hydrolysis
Abundance
Time-->
Rha1
Rha2
GlcNAc
Inos
NAMA1
C
MGAS5005 strong hydrolysis
Abundance
Time-->
Rha1
Rha2
GlcNAc
Inos
NAMA1
NAMA2
NAMA3
D
5005Δ*spyB* strong hydrolysis
Abundance
Time-->
Rha1
Rha2
GlcNAc
Inos
NAMA1
NAMA2
NAMA3

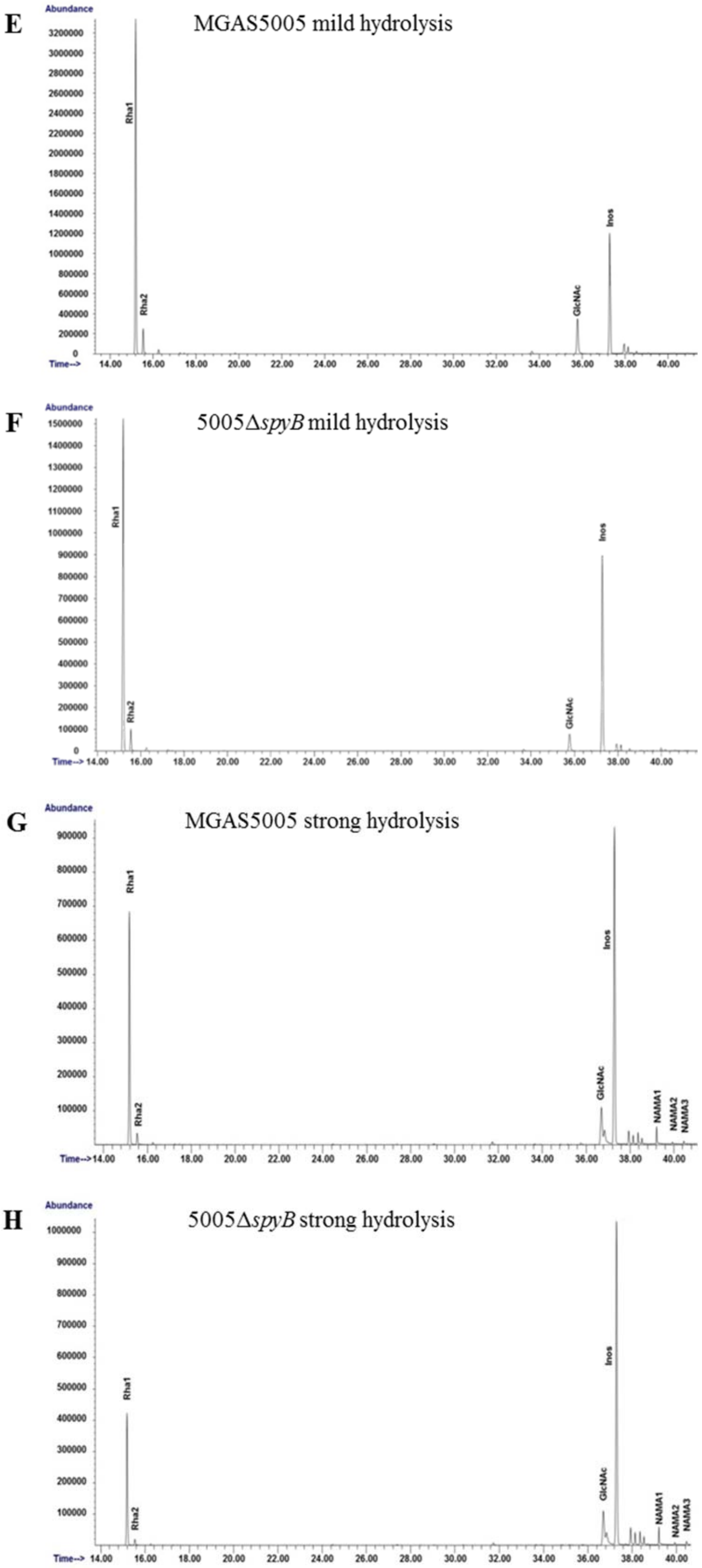
E
MGAS5005 mild hydrolysis
Abundance
Rha1
Rha2
GlcNAc
Inos
Time-->
F
5005Δ*spyB* mild hydrolysis
Abundance
Rha1
Rha2
GlcNAc
Inos
Time-->
G
MGAS5005 strong hydrolysis
Abundance
Rha1
Rha2
GlcNAc
Inos
NAMA1
NAMA2
NAMA3
Time-->
H
5005Δ*spyB* strong hydrolysis
Abundance
Rha1
Rha2
GlcNAc
Inos
NAMA1
NAMA2
NAMA3
Time-->
14.00
16.00
18.00
20.00
22.00
24.00
26.00
28.00
30.00
32.00
34.00
36.00
38.00
40.00

Figure S4. GC-MS chromatograms for glycosyl composition analysis of MGAS5005 and 5005 Δ*spyB*. (**A**) and (**E**) MGAS5005 mild hydrolysis, (**B**) and (**F**) 5005Δ*spyB* mild hydrolysis, (**C**) and (**G**) MGAS5005 strong hydrolysis, (**D**) and (**H**) 5005Δ*spyB* strong hydrolysis. (**A-D)** and (**E-H)** are data from two separate analyses performed on different cell wall preparations.

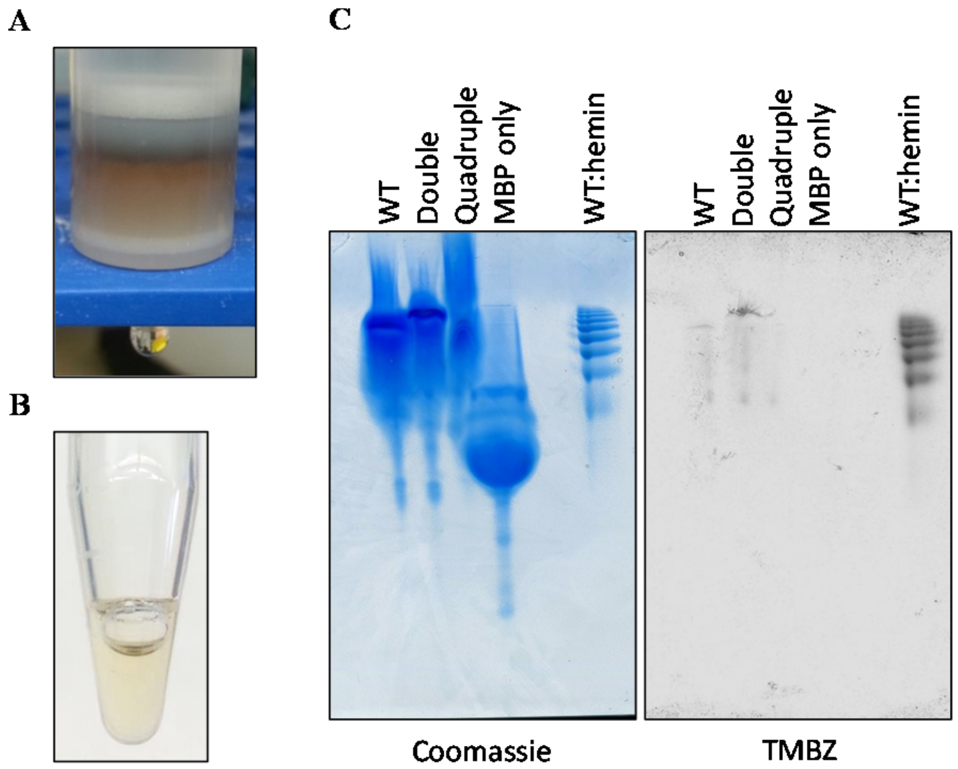

Figure S5. MBP-SpyB binds heme. Images of MBP-SpyB (**A**) eluting from a Ni-NTA column following expression in *E. coli* Rosetta DE3, and (**B**) a sample of the eluate. (**C**) WT MBP-SpyB, double (C7A/C13A) and quadruple (C7A/C13A/C30A/C35A) mutants, MBP and MBP-SpyB reconstituted with hemin were analyzed by native-PAGE following Ni-NTA purification. The native PAGE was stained with coomassie blue and TMBZ-H_2O_2 for heme detection.

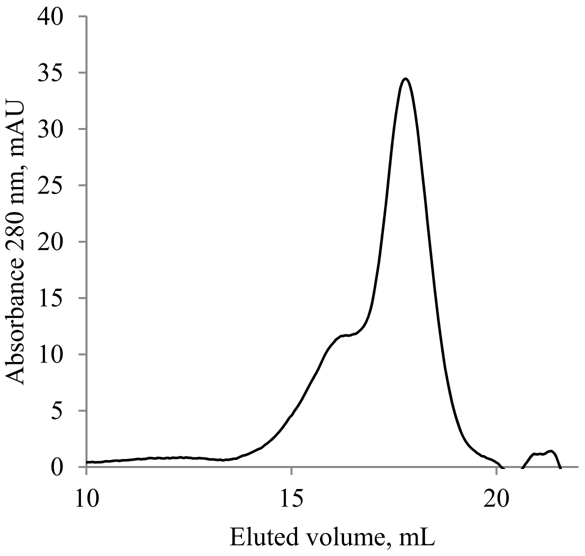

Figure S6. The size-exclusion chromatogram of MBP-SpyB, as the last step of purification, following expression in *E. coli* and subsequent purification by Ni-NTA affinity chromatography.

A

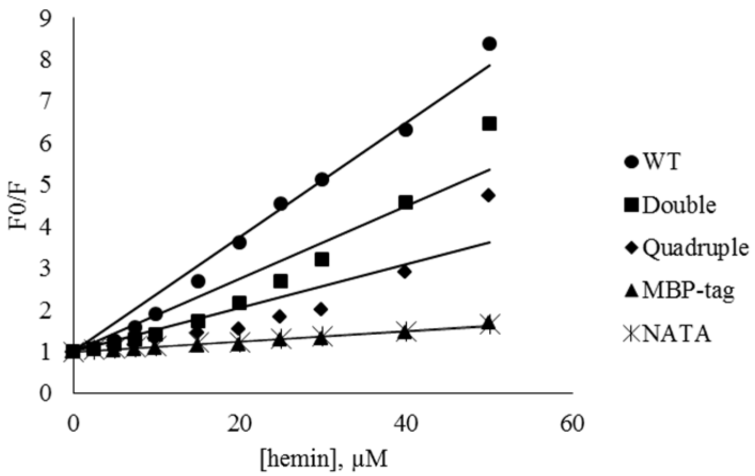

B

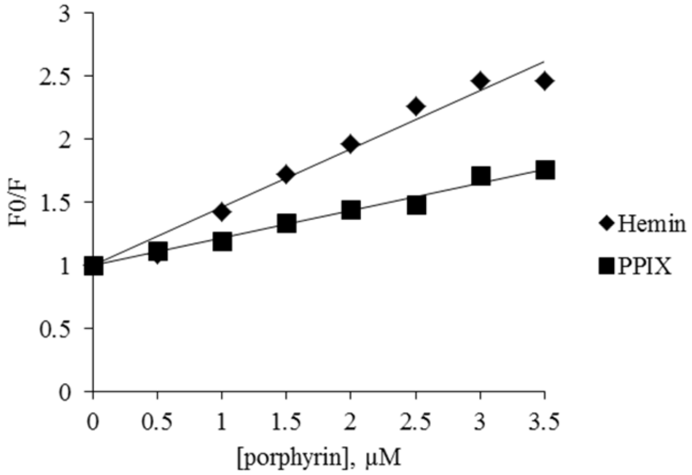

C

	K_{sv} (M^{-1})	R^2
MBP-SpyB	$1.37\text{x}10^{-7}$	0.981
Double	$8.75\text{x}10^{-8}$	0.906
Quadruple	$5.26\text{x}10^{-8}$	0.816
MBP-tag	$1.18\text{x}10^{-8}$	0.953
NATA	$1.28\text{x}10^{-8}$	0.992
SpyB:hemin	$4.26\text{x}10^{-7}$	0.974
SpyB:PPIX	$2.19\text{x}10^{-7}$	0.986

Figure S7. SpyB shows dynamic binding of hemin. (**A**) Stern-Volmer plot of fluorescence quenching by hemin for WT MBP-SpyB, double (7A/13A) and quadruple (7A/13A/30A/35A) mutants. Controls of MBP and *N*-acetyltryptophanamide (NATA) are also shown. (**B**) Stern-Volmer plot of SpyB fluorescence quenching by hemin and protoporphyrin IX (PPIX). (**C**) Table of Stern-Volmer quenching constants calculated according to the equation: $F_0/F=K_{sv}[Q]+1$. F_0 and F are fluorescence intensities in the absence and presence of hemin, K_{sv} is the Stern-Volmer quenching constant and [Q] is the concentration of quencher (hemin or protoporphyrin).

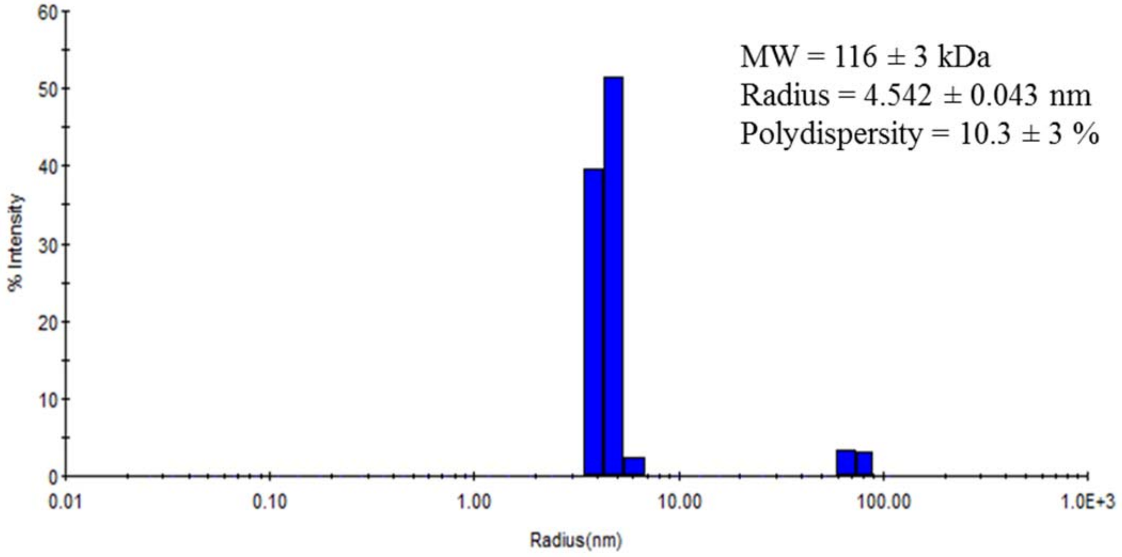

Figure S8. Dynamic light scattering analysis of the oligomer peak from size exclusion chromatography of MBP-SpyB. Dynamic light scattering measurements of 40 μM MBP-SpyB were taken over a 54 s time period with 10 replicates at room temperature. The associated DYNAMICS analysis software was used to determine the average molecular weight, radii and polydispersity (a score < 20% indicates the sample is monodisperse).

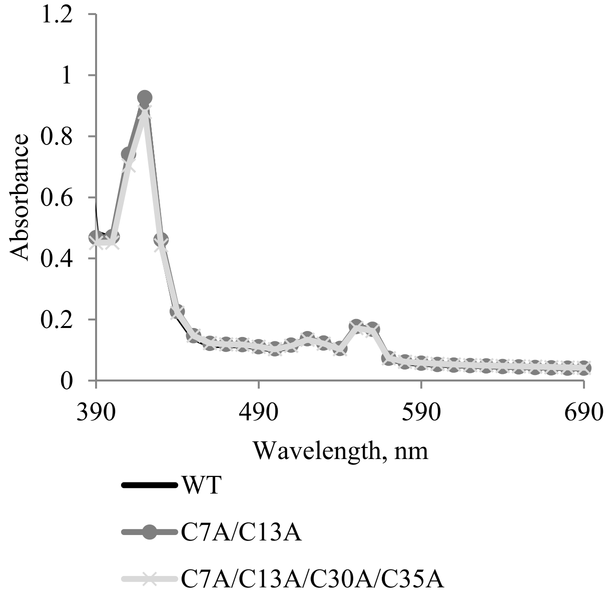

Figure S9. Spectra of reduced pyridine hemochrome derived from WT MPB-SpyB, double (C7A/C13A) and quadruple (C7A/C13A/C30A/C35A) mutants.

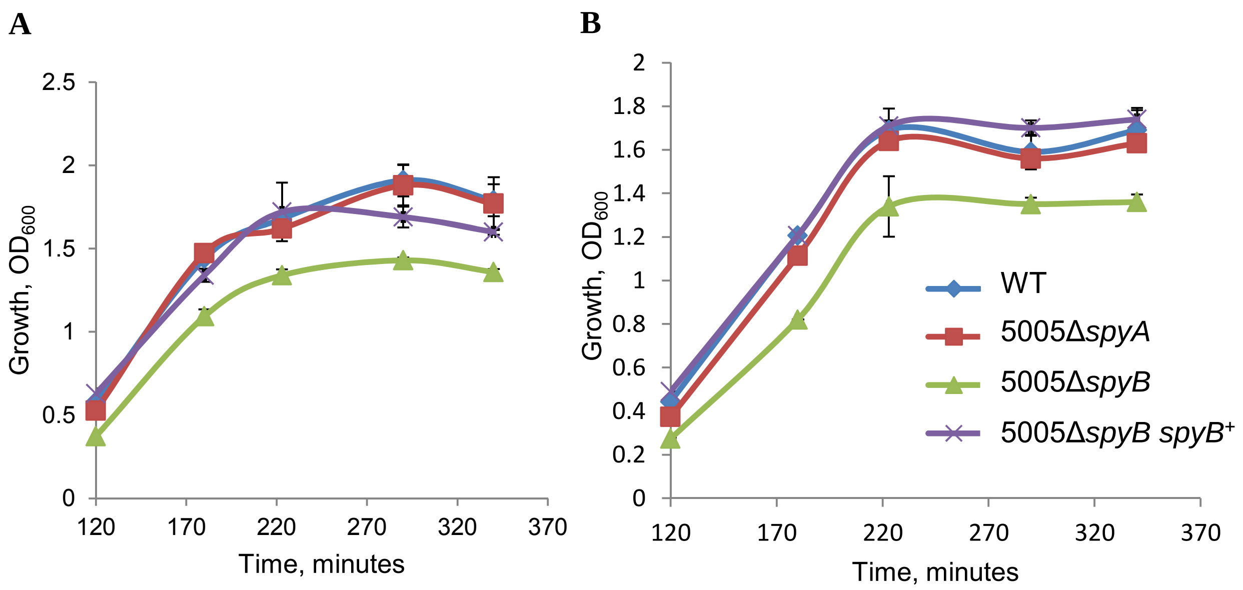

Figure S10. Growth of the MGAS5005 (WT), 5005Δ*spyB*, 5005Δ*spyB spyB*+ and 5005Δ*spyA* in THY medium in the (**A**) absence and (**B**) presence of 2 μM hemin. The data are the mean of three experiments ± standard deviation.

References

Dunstan, R.A., Heinz, E., Wijeyewickrema, L.C., Pike, R.N., Purcell, A.W., Evans, T.J., Praszkier, J., Robins-Browne, R.M., Strugnell, R.A., Korotkov, K.V., and Lithgow, T. (2013). Assembly of the type II secretion system such as found in Vibrio cholerae depends on the novel Pilotin AspS. *PLoS Pathog* 9**,** e1003117, doi:10.1371/journal.ppat.1003117.

Hoff, J.S., Dewald, M., Moseley, S.L., Collins, C.M., and Voyich, J.M. (2011). SpyA, a C3-like ADP-ribosyltransferase, contributes to virulence in a mouse subcutaneous model of Streptococcus pyogenes infection. *Infection and immunity* 79**,** 2404-2411, doi:10.1128/IAI.01191-10.

Moll, I., Grill, S., Gualerzi, C.O., and Blasi, U. (2002). Leaderless mRNAs in bacteria: surprises in ribosomal recruitment and translational control. *Mol Microbiol* 43**,** 239-246.

Sumby, P., Porcella, S.F., Madrigal, A.G., Barbian, K.D., Virtaneva, K., Ricklefs, S.M., Sturdevant, D.E., Graham, M.R., Vuopio-Varkila, J., Hoe, N.P., and Musser, J.M. (2005). Evolutionary origin and emergence of a highly successful clone of serotype M1 group a Streptococcus involved multiple horizontal gene transfer events. *J Infect Dis* 192**,** 771-782.

Zhu, H., Liu, M., Sumby, P., and Lei, B. (2009). The secreted esterase of group a streptococcus is important for invasive skin infection and dissemination in mice. *Infection and immunity* 77**,** 5225-5232, doi:10.1128/IAI.00636-09.
